# Complement C1q-induced activation of β-catenin signalling causes hypertensive arterial remodelling

**DOI:** 10.1038/ncomms7241

**Published:** 2015-02-26

**Authors:** Tomokazu Sumida, Atsuhiko T. Naito, Seitaro Nomura, Akito Nakagawa, Tomoaki Higo, Akihito Hashimoto, Katsuki Okada, Taku Sakai, Masamichi Ito, Toshihiro Yamaguchi, Toru Oka, Hiroshi Akazawa, Jong-Kook Lee, Tohru Minamino, Stefan Offermanns, Tetsuo Noda, Marina Botto, Yoshio Kobayashi, Hiroyuki Morita, Ichiro Manabe, Toshio Nagai, Ichiro Shiojima, Issei Komuro

**Affiliations:** 1 Department of Cardiovascular Medicine, The University of Tokyo Graduate School of Medicine, Tokyo 113-8655, Japan; 2 CREST, Japan Science and Technology Agency, Tokyo 102-0075, Japan; 3 Department of Cardiovascular Medicine, Osaka University Graduate School of Medicine, Osaka 565-0871, Japan; 4 Department of Cardiovascular Biology and Medicine, Niigata University Graduate School of Medical and Dental Sciences, Niigata 951-8510, Japan; 5 Department of Pharmacology, Max-Planck-Institute for Heart and Lung Research, Bad Nauheim D-61231, Germany; 6 Department of Cell Biology, The Cancer Institute, Japanese Foundation for Cancer Research, Tokyo 135-8550, Japan; 7 Centre for Complement and Inflammation Research, Department of Medicine, Imperial College London, London SW7 2AZ, UK; 8 Department of Cardiovascular Medicine, Chiba University Graduate School of Medicine, Chiba 260-8670, Japan; 9 Department of Medicine II, Kansai Medical University, Osaka 573-1191, Japan

## Abstract

Hypertension induces structural remodelling of arteries, which leads to arteriosclerosis and end-organ damage. Hyperplasia of vascular smooth muscle cells (VSMCs) and infiltration of immune cells are the hallmark of hypertensive arterial remodelling. However, the precise molecular mechanisms of arterial remodelling remain elusive. We have recently reported that complement C1q activates β-catenin signalling independent of Wnts. Here, we show a critical role of complement C1-induced activation of β-catenin signalling in hypertensive arterial remodelling. Activation of β-catenin and proliferation of VSMCs were observed after blood-pressure elevation, which were prevented by genetic and chemical inhibition of β-catenin signalling. Macrophage depletion and *C1qa* gene deletion attenuated the hypertension-induced β-catenin signalling, proliferation of VSMCs and pathological arterial remodelling. Our findings unveil the link between complement C1 and arterial remodelling and suggest that C1-induced activation of β-catenin signalling becomes a novel therapeutic target to prevent arteriosclerosis in patients with hypertension.

## Abstract (web summary)

The role of macrophages in hypertension-induced arterial remodeling is poorly understood. Here, Sumida *et al*. show that high blood pressure drives the alternatively activated macrophages to secrete complement C1q protein, which in turn elicits proliferative β-catenin signalling in the arterial smooth muscle cells.

## Graphical abstract

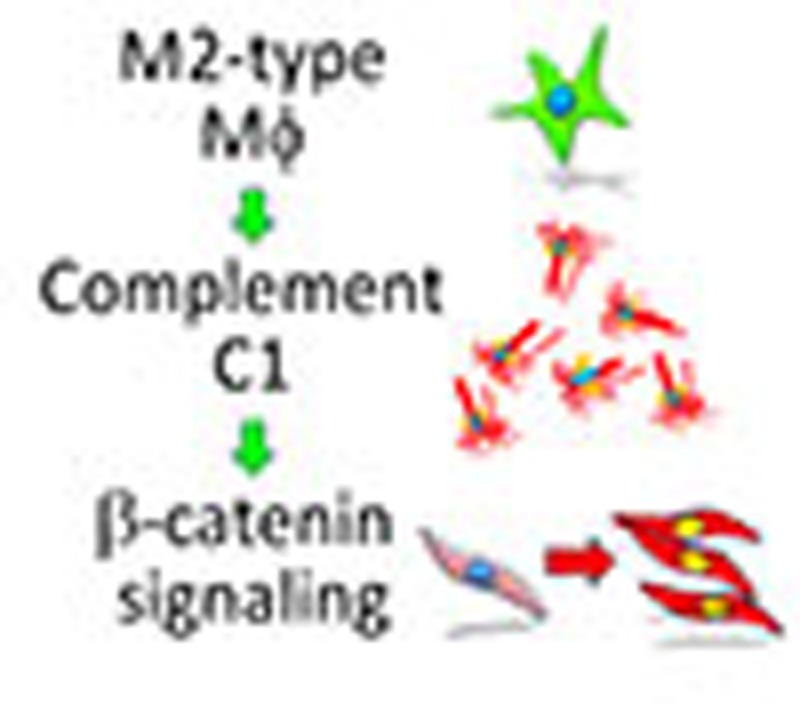

Hypertension, as the leading risk factor for various cardiovascular diseases, caused 9.4 million deaths in 2010 (ref. 1). In 2000, nearly one billion people have hypertension worldwide, and that number is estimated to increase to 1.5 billion by 2025 (ref. 2). One direct physiological consequence of blood-pressure elevation is the structural remodelling of the arteries. Prolonged high blood pressure causes arterial degeneration due to lack of capability of optimized remodelling, leading to pathological conditions such as arteriosclerosis and end-organ damage^3^,^4^. Hypertrophy/hyperplasia of vascular smooth muscle cells (VSMCs) and infiltration of inflammatory cells are the characteristics of pathological arterial remodelling^4^,^5^,^6^. A variety of humoral factors such as growth factors, proteases and cytokines, secreted by infiltrated immune cells, have been reported to be involved in VSMC proliferation^3^,^7^, however, precise molecular and cellular mechanisms of how hypertensive arterial remodelling is developed remain elusive.

Wnt/β-catenin signalling is an evolutionarily conserved intracellular signalling that plays an important role in embryonic development and various diseases^7^,^8^. The Wnt/β-catenin pathway is the most well-understood signalling cascade initiated by Wnt proteins and acts as a mitogenic signal during the development of multiple organs, and the aberrant activation of Wnt/β-catenin signalling pathway is often associated with cancer^9^,^10^,^11^. Wnt/β-catenin signalling regulates the proliferation and differentiation of smooth muscle cells during embryonic and postnatal angiogenesis^12^,^13^. Furthermore, activation of Wnt/β-catenin signalling is implicated in VSMC proliferation during intimal thickening after vascular injury^14^,^15^.

Recently, we have reported that complement protein C1q, an initiator of the classical complement pathway, activates canonical Wnt signalling in a complement cascade-independent manner^16^. Given that the major source of complement C1q is monocyte-derived cells^17^ and that macrophages (Mφs) within the aortic wall play a vital role in pathogenesis of arterial remodelling, we hypothesized that aortic Mφ-derived C1q activates β-catenin signalling and induces proliferation of VSMCs. In the present study, we elucidated that complement C1q, which is mainly secreted by alternatively activated aortic Mφs, is involved in hypertensive arterial remodelling via activation of β-catenin signalling.

## Results

### VSMC proliferation as an early event in hypertension

**Figure 1:**
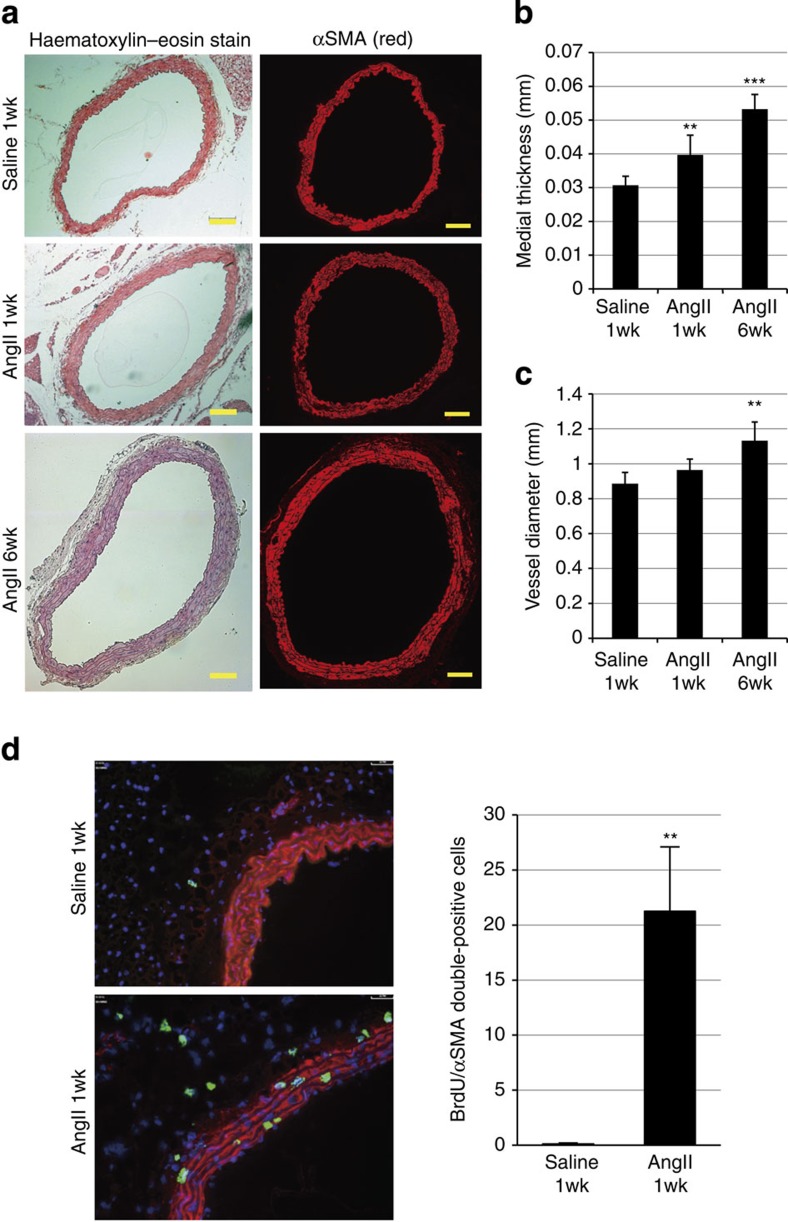
Proliferation of VSMCs is observed at the initial stage of AngII-induced arterial remodelling. (**a**) Haematoxylin and eosin staining and immunostaining for α-smooth muscle actin (αSMA) of the abdominal aorta from 1-week saline-infused mice (saline 1wk), 1-week AngII-infused mice (AngII 1wk) and 6-week AngII-infused mice (AngII 6wk) (*n*=5–7). Scale bar, 100 μm. (**b**,**c**) Morphometric analysis. (**b**) Medial thickness and (**c**) vessel diameter were calculated using ImageJ. **P<0.01, ***P<0.001 versus saline-infused mice (*n*=5–9). (**d**) Aortic tissues were immunostained for BrdU (green) and αSMA (red). Scale bar, 100 μm. The number of double-positive (BrdU(+)/αSMA(+)) cells per section is shown. **P<0.01 versus saline-infused mice (*n*=5–7). Statistical significance was determined using one-way analysis of variance with Turkey’s *post hoc* test for **b** and **c**, and the unpaired two-tailed Student’s *t*-test for **d**. Results are represented as mean±s.d.

Infusion of angiotensin II (AngII) (1.8 μg kg^−1^ min^−1^) to male C57BL/6 mice increased blood pressure by ~70 mm Hg and promoted arterial remodelling characterized by thickening and dilation of the abdominal aorta at 6 weeks after AngII infusion (Fig. 1a–c), as reported previously^18^. The number of 5-bromo-2′deoxyuridine (BrdU)-positive, proliferating VSMCs was significantly increased as early as 1 week after AngII infusion (Fig. 1d), when the gross structural remodelling of the arteries was not observed. These results suggest that VSMC proliferation is one of the earliest events that occur in response to AngII-induced blood-pressure elevation.

### β-catenin signalling is activated at early hypertension

Various growth factors and G-protein-coupled receptor agonists induce VSMC proliferation^7^,^19^, and their mitogenic effects are often given by the activation of the mitogen-activated protein kinases, especially the extracellular signal regulated kinase (ERK) signalling pathway^5^,^20^. Therefore, we first examined whether ERK signalling was activated concurrently with VSMC proliferation after AngII infusion, and found that there was no increase in the phosphorylation levels of ERK1/2 (Supplementary Fig. 1a). Moreover, AngII activated ERK1/2 but had no effect on proliferation of cultured human aortic smooth muscle cells (HASMCs) (Supplementary Fig. 1b,c), suggesting that signalling pathways other than the ERK pathway play a key role in VSMC proliferation at the early stage of AngII-induced hypertension.

**Figure 2:**
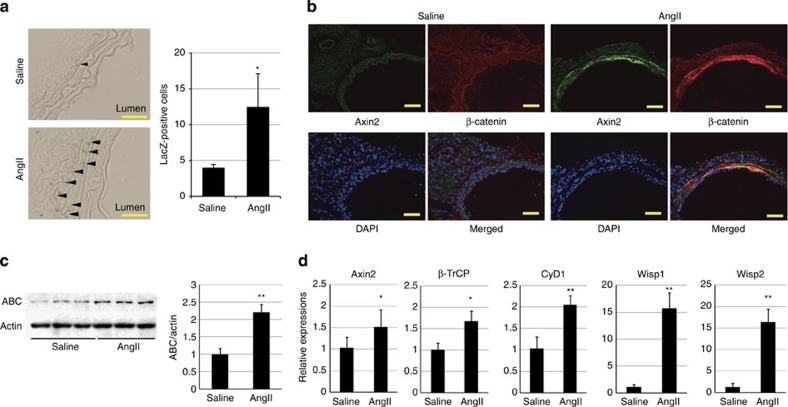
β-catenin signalling is activated in the aortic media at the early stage of hypertension. (**a**) β-galactosidase staining of the aortic tissue from 1-week saline- or AngII-infused Axin2^LacZ^ mice. Arrowheads indicate β-galactosidase-positive nuclei. Scale bar, 50 μm. The number of LacZ-positive cells in the aortic media from Axin2^LacZ^ mice is shown. *P<0.05 versus saline-infused mice (*n*=5). (**b**) Aortic tissues from 1-week saline- or AngII-infused mice were immunostained for Axin2 (green) and β-catenin (red). Scale bar, 100 μm. (**c**) Western blot analysis for non-phosphorylated active β-catenin (ABC) in the aortic tissues from 1-week saline- or AngII-infused mice. Activation of β-catenin signalling was quantified by measuring the relative level of ABC over actin. The values are shown as fold induction over saline-infused mice. **P<0.01 versus saline-infused mice (*n*=6, 7). (**d**) Real-time PCR analysis for the expression levels of the β-catenin target genes (*Axin2*, *β-TrCP*, *cyclin D1 (CyD1)*, *Wisp1* and *Wisp2*) in the aortic tissue from 1-week saline- or AngII-infused mice. The values are shown as fold induction over saline-infused mice. *P<0.05, **P<0.01 versus saline (*n*=6). Statistical significance was determined using the unpaired two-tailed Mann–Whitney *U*-test for **a**, **c** and **d**. Results are represented as mean±s.d. DAPI, 4',6-diamidino-2-phenylindole.

Wnt/β-catenin signalling has been reported to regulate proliferation and differentiation of smooth muscle cells during embryonic and postnatal angiogenesis^12^,^13^ and intimal thickening after vascular injury^14^,^15^. We therefore tested whether β-catenin signalling was involved in VSMC proliferation after AngII infusion. Activation of β-catenin signalling upregulates the expression of Wnt/β-catenin target genes including *Axin2* (ref. 10). At 1 week after AngII infusion, the number of LacZ-positive VSMCs was increased in Wnt reporter Axin2^LacZ^ mice^11^ (Fig. 2a) and the aortic VSMCs were strongly stained for β-catenin and Axin2 in wild-type mice (Fig. 2b; Supplementary Fig. 2a,b). Western blot (WB) analysis revealed that the amount of non-phosphorylated, active β-catenin (ABC)^21^ was increased (Fig. 2c), and gene expression analysis also showed upregulation of Wnt/β-catenin target genes in aortic tissue (Fig. 2d). Although β-catenin was also expressed in endothelial cells of aorta (Supplementary Fig. 2c), Axin2 expression was not induced in endothelial cells 1 week after AngII infusion (Fig. 2a,b; Supplementary Fig. 2b), suggesting that β-catenin signalling was more potently activated in aortic VSMCs rather than endothelial cells at the early stage of hypertension. Both activation of β-catenin signalling and proliferation of VSMCs were attenuated when we normalized blood pressure in AngII-infused mice by oral administration of hydralazine (Supplementary Fig. 3a–c). Taken together, these results suggested that the elevation of blood pressure was responsible in part for activation of β-catenin signalling in the aortic VSMCs during hypertensive arterial remodelling.

### β-catenin signal activation induces VSMC proliferation

**Figure 3:**
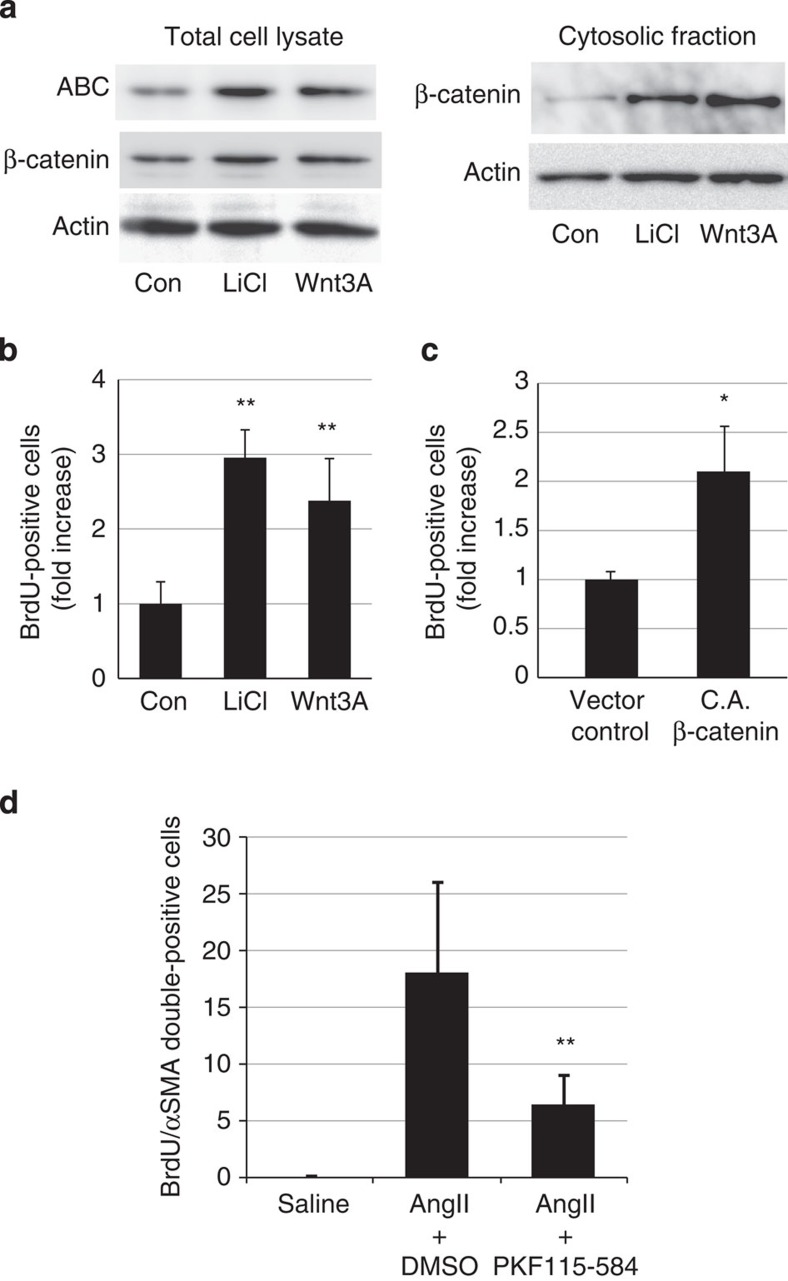
Activation of β-catenin signalling induces VSMC proliferation. (**a**) Western blot analysis. HASMCs were treated with LiCl (10 mM) or Wnt3A (80 ng ml^−1^), and the amount of ABC and cytosolic β-catenin was analysed. Activation of β-catenin signalling was quantified by measuring the relative level of ABC over actin. (**b**) The number of BrdU(+) HASMCs after LiCl (10 mM) or Wnt3A (80 ng ml^−1^) treatment. The values are shown as fold induction over non-treated HASMCs (Con). **P<0.01 versus non-treated HASMCs (*n*=4). (**c**) The number of BrdU(+) HASMCs after infection with control retrovirus (vector control) or with constitutively active β-catenin (CA β-catenin) whose phosphorylation sites at the N terminus are all mutated. The values are shown as fold induction over control vector-transfected HASMCs. *P<0.05 versus control vector-transfected HASMCs (*n*=3). (**d**) The number of double-positive (BrdU(+)/αSMA(+)) cells per aortic section from saline-infused mice, AngII-infused mice treated with DMSO (solvent), and AngII-infused mice treated with PKF115-584. **P<0.01 versus AngII-infused mice treated with DMSO (*n*=8). Statistical significance was determined using one-way analysis of variance with Turkey’s *post hoc* test for **b**, the unpaired two-tailed Student’s *t*-test for **c** and the Kruskal–Wallis test with Dunn’s correction for multiple comparisons for **d**. Results are represented as mean±s.d.

**Figure 4:**
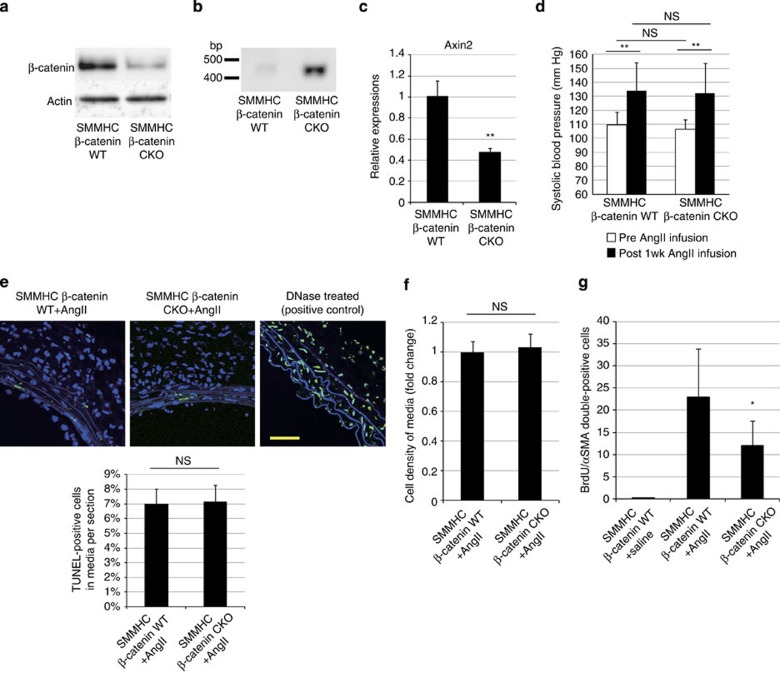
β-catenin signal activation is responsible for VSMC proliferation after AngII infusion. (**a**) Western blot analysis. The amounts of β-catenin in the aortic tissues from SMMHC-CreER^T2^:*Ctnnb1*^+/+^ mice (SMMHC-β-catenin wild type (WT)) and SMMHC-CreER^T2^:*Ctnnb1*^flox/flox^ mice (SMMHC-β-catenin CKO) were analysed in aortic tissues isolated 6 days after the final tamoxifen treatment. (**b**) PCR analysis of aortic tissue DNA. DNA extracted from aortic tissues of tamoxifen-treated SMMHC-β-catenin WT and SMMHC-β-catenin CKO mice were amplified with a PCR primer set designed for detecting the null allele. (**c**) Real-time PCR analysis for the expression level of the *Axin2* gene (one of the major Wnt/β-catenin target genes) in the aortic tissue isolated from tamoxifen-treated SMMHC-β-catenin WT and SMMHC-β-catenin CKO mice. The values are shown as fold induction over SMMHC-β-catenin WT mice. **P<0.01 versus SMMHC-β-catenin WT mice. (**d**) Systolic blood pressure before and after AngII infusion for 1 week. There was no difference in systolic blood pressure between SMMHC-β-catenin WT mice and SMMHC-β-catenin CKO mice before and after AngII infusion. **P<0.01 versus Post 1wk AngII infusion. (**e**) TdT-mediated dUTP nick end labelling (TUNEL) staining of aortic tissue and percentage of TUNEL-positive cells. TUNEL staining of aortic tissue from SMMHC-β-catenin WT and SMMHC-β-catenin CKO mice 1 week after AngII infusion. The DNase (TACS nuclease)-treated section is presented as a positive control. Percentage of TUNEL-positive cells per total cells in aortic media was calculated. Scale bar, 50 μm. (**f**) Cell density of aortic media was calculated by measuring the number of αSMA-positive cells per field of view size (40 × 40 μm^2^). (**g**) The number of double-positive (BrdU(+)/αSMA(+)) cells per aortic section from 1-week saline-infused SMMHC-CreER^T2^:*Ctnnb1*^+/+^ mice (SMMHC-β-catenin WT+saline), 1-week AngII-infused SMMHC-CreER^T2^:*Ctnnb1*^+/+^ mice (SMMHC-β-catenin WT+AngII) and SMMHC-CreER^T2^:*Ctnnb1*^flox/flox^ mice (SMMHC-β-catenin CKO+AngII). *P<0.05 versus SMMHC-β-catenin WT+AngII (*n*=12). The values are shown as fold induction over SMMHC-β-catenin WT mice (*n*=5). Statistical significance was determined using the unpaired two-tailed Student’s *t*-test for **c**, **e** and **f**, two-way analysis of variance followed by Sidak’s multiple comparisons test for **d** and the Kruskal–Wallis test with Dunn’s correction for multiple comparisons for **g**. Results are represented as mean±s.d. NS, not significant.

To determine whether the activation of β-catenin signalling induces proliferation of VSMCs, we first treated cultured HASMCs with Wnt3A, a canonical Wnt ligand, or lithium chloride, which stabilizes β-catenin by inhibiting glycogen synthase kinase-3. Both Wnt3A and lithium chloride treatment activated β-catenin signalling and induced proliferation of HASMCs (Fig. 3a,b). Moreover, overexpression of constitutively ABC also induced proliferation of HASMCs (Fig. 3c), suggesting that activation of β-catenin signalling is sufficient to induce VSMC proliferation *in vitro*. On the other hand, AngII treatment did not induce activation of β-catenin signalling or proliferation of HASMCs (Supplementary Figs 1c and 3d), but induced cellular hypertrophy of HASMCs (Supplementary Fig. 3e). We next examined whether activation of β-catenin signalling was required for AngII-induced VSMC proliferation *in vivo*. Intraperitoneal injection of PKF115-584, a small-molecule inhibitor of β-catenin signalling^22^, suppressed VSMC proliferation without lowering blood pressure at 1 week after AngII infusion (Fig. 3d; Supplementary Fig. 3f). To further determine the role of the β-catenin signal in VSMCs, we generated smooth muscle cell-specific, tamoxifen-inducible, β-catenin knockout mice by crossing SMMHC-CreER^T2^ mice^23^ with *Ctnnb1*^flox/flox^ mice (SMMHC/β-catenin CKO). The expression levels of β-catenin and Axin2 in aortic tissue were downregulated when they were treated with tamoxifen (Fig. 4a–c). There was no change in systolic blood pressure of SMMHC/β-catenin CKO mice as compared with control mice (Fig. 4d). TdT-mediated dUTP nick end labelling staining revealed that there was no change in cell death and cell density in aortic media after tamoxifen treatment (Fig. 4e,f). There was less proliferation of VSMCs after AngII infusion in SMMHC/β-catenin CKO mice as compared with control mice (Fig. 4g), suggesting that activation of β-catenin signalling substantially contributes to enhanced proliferation of VSMCs at the early phase of hypertensive arterial remodelling.

### Recruitment of Mφs is essential for β-catenin signalling

**Figure 5:**
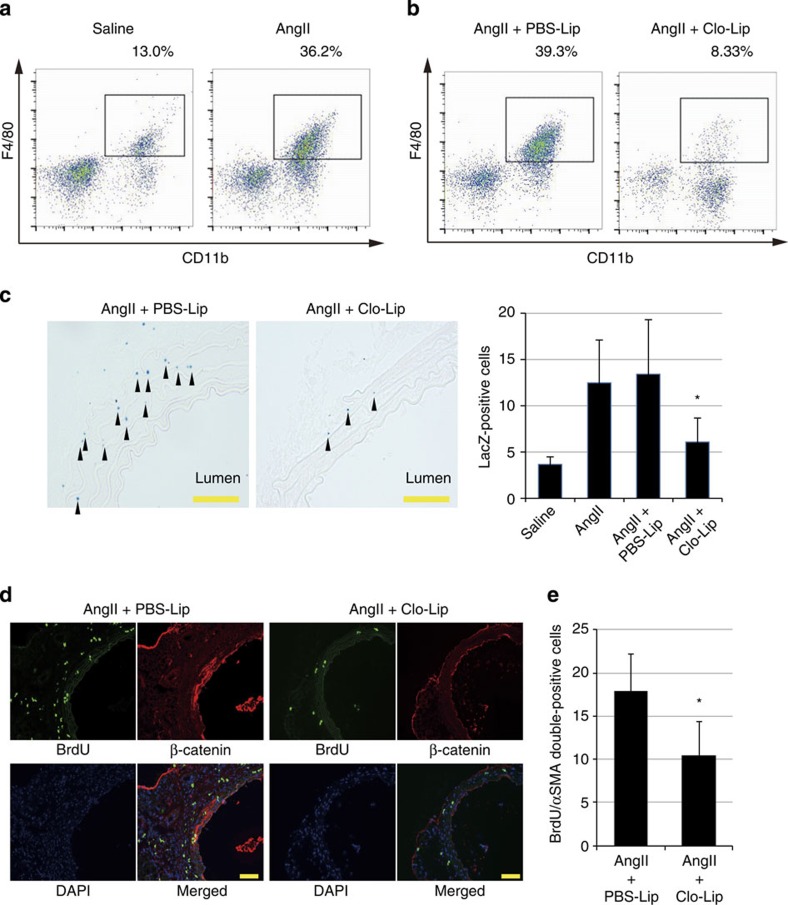
Recruited Mφs activate β-catenin signalling and induce VSMC proliferation after AngII infusion. (**a**,**b**) Representative density plots. Aortic Mφs of 1-week saline- and AngII-infused mice (**a**) of and in AngII-infused mice treated with PBS liposome (PBS-Lip) or clodronate liposome (Clo-Lip) (**b**) were analysed by flow cytometry. Cells within the boxes are CD11b+F4/80+Mφs. The flow cytometric analysis was performed with pooled aortic tissues from a total of 3–10 mice and percent gated cell frequencies are indicated in each representative plot. (**c**) β-Galactosidase staining of the aortic tissue from AngII-infused Axin2^LacZ^ mice, treated with PBS-Lip or Clo-Lip. Arrowheads indicate β-galactosidase-positive nuclei. Scale bar, 50 μm. The number of LacZ-positive cells in the media of aortic tissue from Axin2^LacZ^ mice is shown. *P<0.05 versus PBS-Lip-treated AngII-infused mice (*n*=5–6). (**d**) Aortic tissues from AngII-infused mice treated with PBS-Lip or Clo-Lip were immunostained for BrdU (green) and β-catenin (red). Scale bar, 100 μm. (**e**) The number of double-positive (BrdU(+)/αSMA(+)) cells per aortic section from AngII-infused mice treated with PBS-Lip or Clo-Lip. *P<0.05 versus PBS-Lip-treated AngII-infused mice (*n*=5). Statistical significance was determined using one-way analysis of variance with Turkey’s *post hoc* test for **c**, and the unpaired two-tailed Mann–Whitney *U*-test for **e**. Results are represented as mean±s.d. DAPI, 4',6-diamidino-2-phenylindole.

Mφs were recruited to the aortic adventitia by 1 week after AngII infusion (Fig. 5a; Supplementary Fig. 4a). To elucidate the role of these Mφs, we injected liposomes containing clodronate (Clo-Lip)^24^ before AngII infusion to deplete the cells of monocyte lineage. Clo-Lip treatment reduced the number of infiltrating Mφs (Fig. 5b; Supplementary Fig. 4a), and strongly suppressed activation of β-catenin signalling and proliferation of VSMCs at 1 week after AngII infusion (Fig. 5c–e). These results suggest that infiltrating Mφs play critical roles in activation of β-catenin signalling and proliferation of VSMCs at the early phase of arterial remodelling.

### M2 Mφs secrete a potent activator of β-catenin signalling

**Figure 6:**
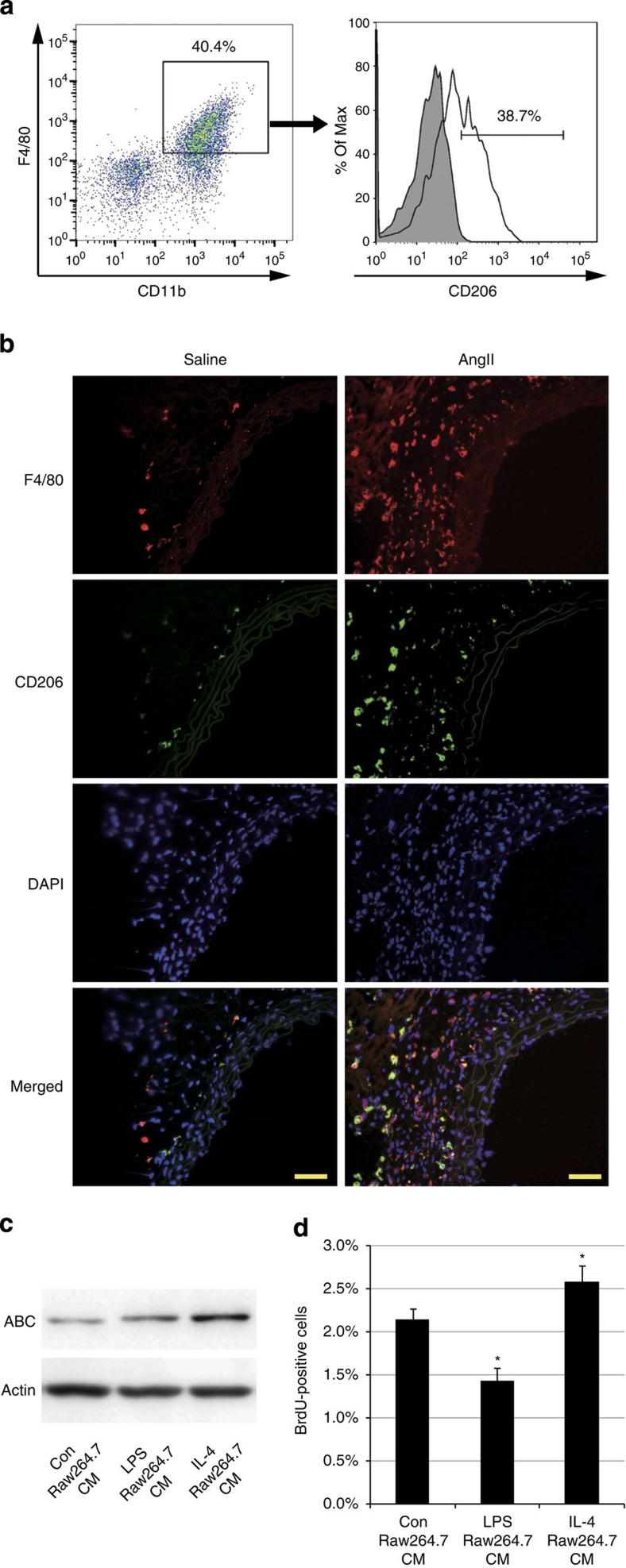
M2-type Mφs are the key players that activate β-catenin signalling during hypertension. (**a**) Representative density plots and histogram. Aortic CD11b+F4/80+ Mφs in AngII-infused mice were further analysed for CD206 positivity. The shaded histogram indicates an isotype-control stained sample. (**b**) Aortic tissues from saline- or AngII-infused mice were immunostained for CD206 (green) and F4/80 (red). Scale bar, 50 μm. (**c**) Representative western blot analysis. Conditioned media from Raw264.7 cells treated with PBS (Con Raw264.7 CM), LPS (50 ng ml^−1^) (LPS Raw264.7 CM) or IL-4 (20 ng ml^−1^) (IL-4 Raw264.7 CM) were added to HASMCs, and the amount of ABC in the total cell lysate of HASMCs was analysed. (**d**) HASMCs were treated as in **c**, and the percentage of BrdU-positive cells was counted. *P<0.05 versus Con Raw264.7 CM (*n*=4). Statistical significance was determined using one-way analysis of variance with Turkey’s *post hoc* test for **d**. Results are represented as mean±s.d. DAPI, 4′,6-diamidino-2-phenylindole.

Mφs change their phenotypes in response to various environmental factors. Classically activated Mφs or M1 Mφs are induced by lipopolysaccharide (LPS), interferon-γ and tumour necrosis factor, and secrete pro-inflammatory cytokines to increase their killing ability. On the other hand, alternatively activated Mφs or M2 Mφs are induced by interleukin (IL)-4 and IL-13, and contribute to tissue remodelling and wound healing^25^. To determine the phenotype of Mφs that accumulate into the aortic wall at the early stage of arterial remodelling, we used flow cytometric analysis to characterize M1-type Mφ for Ly6c expression and M2-type Mφ for CD206 expression^26^. Approximately 40% of the aortic Mφs from AngII-infused mice expressed CD206 (CD11b+F4/80+CD206+Ly6c−: M2 type) and ~10% of them expressed Ly6c (CD11b+F4/80+CD206-Ly6c+: M1 type) (Fig. 6a; Supplementary Fig. 4b). Immunofluorescent analysis also showed large numbers of CD206-positive Mφs in aortic adventitia (Fig. 6b). These results prompted us to postulate that M2-type Mφs secrete humoral factors that activate β-catenin signalling and induce proliferation of VSMCs. To test this hypothesis, we first examined the effects of humoral factors secreted by M2 Mφs on VSMCs. The M1 or M2 phenotype was elicited *in vitro* by treating Raw264.7 cells with LPS or IL-4, respectively. Conditioned media (CM) from Raw264.7 cells treated with PBS (Con Raw264.7 CM), LPS (LPS Raw264.7 CM) or IL-4 (IL-4 Raw264.7 CM) were added to HASMCs. IL-4 Raw264.7 CM activated β-catenin signalling and promoted cell proliferation in HASMCs more potently than Con Raw264.7 CM or LPS Raw264.7 CM (Fig. 6c,d). As Raw264.7 cells are known to be already activated, we performed the same experiments using bone marrow-derived Mφs (BMDMs), and found that CM from IL-4-treated BMDMs (IL-4 BMDM CM) activated β-catenin signalling and induced proliferation of VSMCs more than CM from PBS-treated BMDMs (Con BMDM CM) or LPS-treated BMDMs (LPS BMDM CM) (Supplementary Fig. 4c,d). These results collectively suggest that M2 Mφs, recruited to the vessel wall in response to blood-pressure elevation, secrete a substance that activates β-catenin signalling in VSMCs, thereby inducing VSMC proliferation in a paracrine manner. Expression levels of canonical Wnt ligands (Wnt1, 2, 2b,3, 3a, 7a, 7b, 8a, 8b, 10a and 10b), which could activate the β-catenin signalling pathway^10^,^27^,^28^,^29^, were not upregulated in Raw264.7 cells or BMDMs by IL-4-induced M2 polarization (Supplementary Fig. 4e,f).

### Complement C1q is a Mφ-derived β-catenin signal activator

**Figure 7:**
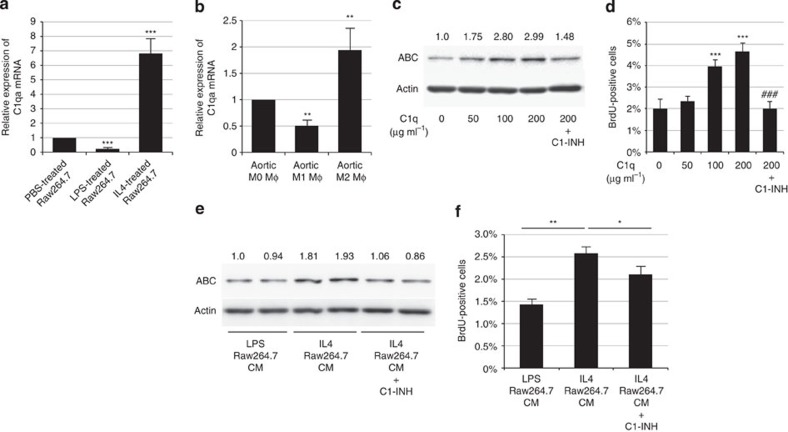
C1q secreted from M2-type Mφs activates β-catenin signalling and induces VSMC proliferation. (**a**) Real-time PCR analysis for the expression level of *C1qa* gene in Raw264.7 cells treated with PBS, LPS (50 ng ml^−1^) or IL-4 (20 ng ml^−1^). The values are shown as fold induction over PBS-treated Raw264.7 cells. ***P<0.001 versus PBS-treated Raw264.7 cells (*n*=4). (**b**) M0, M1 and M2 Mφs from 10 pooled aortic tissues from AngII-infused mice were sorted by flow cytometry and the expression level of *C1qa* gene was analysed by real-time PCR. The values are shown as fold induction over aortic M0 Mφs (*n*=4). (**c**) Representative western blot analysis. HASMCs were treated with C1q (50, 100 and 200 μg ml^−1^) and C1-INH (150 μg ml^−1^). The protein amount of ABC was analysed and the relative intensity of each band is shown over each immunoblot after normalization for the level of actin. (**d**) HASMCs were treated as in **c**, and the number of BrdU-positive cells was counted. ***P<0.001 versus non-treated cells (C1q 0 μg ml^−1^) (*n*=4), ^###^*P*<0.001 versus C1q (200 μg ml^−1^) treated cells. (**e**) Representative western blot analysis. Conditioned media from Raw264.7 cells treated with LPS (50 ng ml^−1^) (LPS Raw264.7 CM) or IL-4 (20 ng ml^−1^) (IL-4 Raw264.7 CM) were added to HASMCs with or without C1-INH (150 μg ml^−1^). The protein amount of ABC in the total cell lysate of HASMCs was analysed and the relative intensity of each band is shown over each immunoblot after normalization for the level of actin. (**f**) HASMCs were treated as in **e**, and the number of BrdU-positive cells was counted (*n*=4). **P<0.01 versus LPS Raw264.7 CM. *P<0.05 versus IL-4 Raw264.7 CM. Statistical significance was determined using one-way analysis of variance with Turkey’s *post hoc* test. Results are represented as mean±s.d. mRNA, messenger RNA.

We have recently reported that complement protein C1q, an initiator of the classical complement pathway, activates β-catenin signalling and induces aging-associated impairment of skeletal muscle regeneration^16^. Given that monocyte/Mφ lineage has been shown to be the major source of C1q biosynthesis^17^ and tumour-associated Mφs have been reported to produce complement C1q^30^, we examined whether β-catenin signalling was activated in VSMCs by M2 Mφ-derived C1q. *In vitro*, M2-polarized Raw264.7 Mφs by IL-4 treatment markedly increased *C1qa* gene expression compared with PBS-treated or LPS-treated Raw264.7 Mφs (Fig. 7a). To examine whether C1q is predominantly produced by M2 Mφs rather than M0 or M1 Mφs *in vivo*, we sorted M0-type (CD11b+F4/80+CD206−Ly6c−), M1-type (CD11b+F4/80+CD206−Ly6c+) and M2-type (CD11b+F4/80+CD206+Ly6c−) Mφs from aortic tissue and compared the expression levels of C1q among three types of Mφs, and found that M2-type Mφs expressed more C1q than M0- or M1-type Mφs (Fig. 7b; Supplementary Fig. 4b).

The direct effect of C1q on HASMCs was examined *in vitro*. C1q protein activated β-catenin signalling and promoted proliferation of cultured HASMCs in a dose-dependent manner, and these effects were suppressed by C1-inhibitor (C1-INH), an endogenous inhibitor of C1r and C1s (Fig. 7c,d; Supplementary Fig. 5a). Moreover, treatment with the C1 complex, which is composed of C1q, C1r and C1s, also activated β-catenin signalling in HASMCs, and this effect was inhibited by C1-INH (Supplementary Fig. 5b). WB analysis of cell culture media revealed that both C1r and C1s were secreted from HASMCs but not from Mφs (Supplementary Fig. 5c,d). Activation of β-catenin signalling and proliferation of HASMCs induced by M2 Mφ-CM was prevented by C1-INH (Fig. 7e,f; Supplementary Fig. 5e,f). These results collectively suggest that the C1 complex, which is composed of M2 Mφ-derived C1q and VSMC-derived C1r/s is the factor that activates β-catenin signalling and induces proliferation of VSMCs.

### C1-induced β-catenin signalling causes arterial remodelling

**Figure 8:**
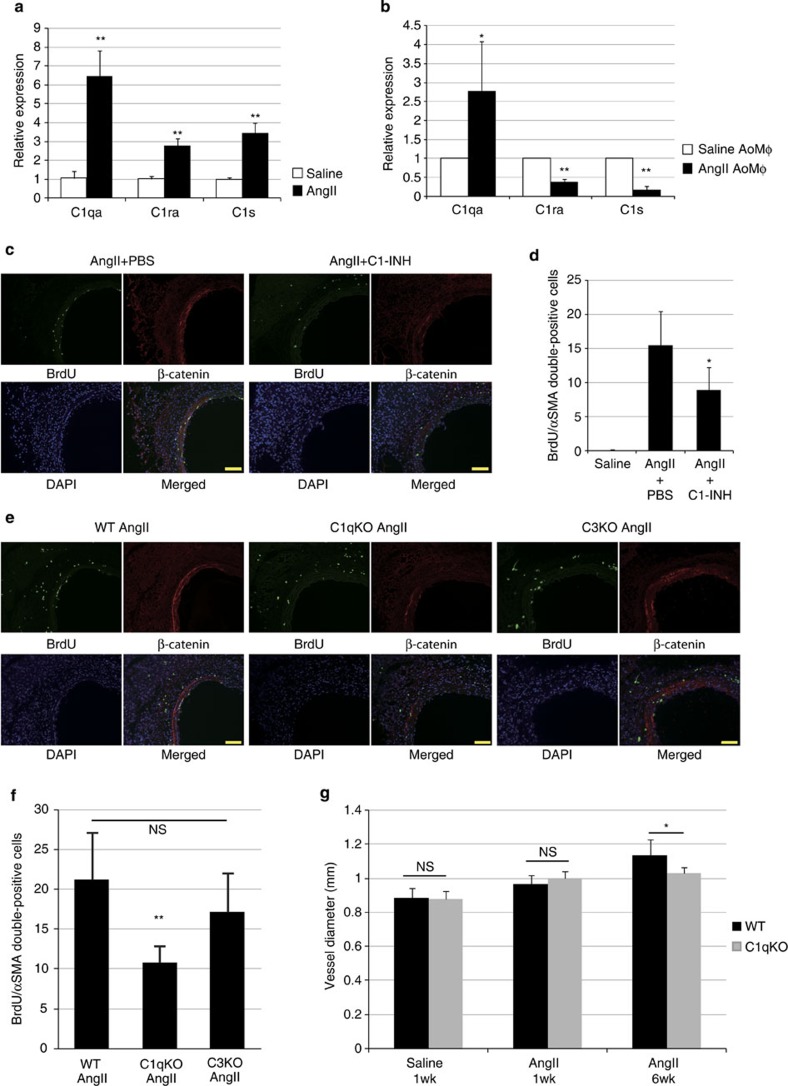
C1q mediates AngII-induced activation of β-catenin signalling and arterial remodelling. (**a**) Real-time PCR analysis for the expression levels of *C1qa*, *C1ra and C1s* genes in the aortic tissue from 1-week saline- or AngII-infused mice. Values are shown as fold induction over saline-infused mice. **P<0.01 versus saline-infused mice (*n*=6). (**b**) Real-time PCR analysis for the expression levels of *C1qa*, *C1ra and C1s* genes in aortic Mφs sorted by flow cytometry from 1-week saline- or AngII-infused mice. The values are shown as fold induction over aortic Mφs isolated from saline-infused mice (saline AoMp). *P<0.05, **P<0.01 versus saline AoMp (*n*=4). (**c**) Aortic tissues from 1-week AngII-infused mice treated with PBS or with C1-INH were immunostained for BrdU (green) and β-catenin (red). Scale bar, 100 μm. (**d**) The number of double-positive (BrdU(+)/αSMA(+)) cells per section. *P<0.05 versus AngII-infused mice treated with PBS (*n*=8). (**e**) Aortic tissues from AngII-infused wild-type mice (WT AngII), C1qa-deficient mice (C1qKO AngII) and C3-deficient (C3KO AngII) mice were immunostained for BrdU (green) and β-catenin (red). Scale bar, 100 μm. (**f**) The number of double-positive (BrdU(+)/αSMA(+)) cells per section. **P<0.01 versus 1-week AngII-infused wild-type mice (*n*=4–7). NS, not significant. (**g**) Morphometric analysis. Aortic tissues from WT mice or C1qKO mice after saline- or AngII-infusion were immunostained for αSMA and the vessel diameter was measured using ImageJ. *P<0.05 versus 6-week AngII-infused WT mice (*n*=5–9). Statistical significance was determined using the unpaired two-tailed Mann–Whitney *U*-test for **a** and **b**, the Kruskal–Wallis test with Dunn’s correction for multiple comparison for **d**, one-way analysis of variance (ANOVA) with Turkey’s *post hoc* test for **f** and the two-way ANOVA followed by Tukey’s multiple comparisons test for **g**. Results are represented as mean±s.d. DAPI, 4′,6-diamidino-2-phenylindole.

We next examined whether C1 is responsible for hypertension-induced activation of β-catenin signalling and proliferation of VSMCs *in vivo*. Expression levels of *C1qa*, *C1ra* and *C1s* genes were increased in the aortic tissue 1 week after AngII infusion (Fig. 8a). Aortic Mφs increased *C1qa* gene expression, but not *C1ra* and *C1s*, 1 week after AngII infusion (Fig. 8b). Treatment with C1-INH prevented the activation of β-catenin signalling in aortic VSMCs and suppressed proliferation of VSMCs induced by AngII infusion (Fig. 8c,d; Supplementary Fig. 6a). In addition, activation of β-catenin signalling and proliferation of VSMCs were both attenuated in C1qa-deficient mice but not in C3-deficient mice at 1 week after AngII infusion (Fig. 8e,f; Supplementary Fig. 6b,c), indicating that these phenotypes induced by C1 are independent of complement cascade activation. There was no significant difference in blood pressure among all groups of mice (Supplementary Fig. 3f). The size of the VSMCs following AngII infusion was comparable between wild-type and C1qa-deficient mice (Supplementary Fig. 6d). Arterial remodelling at 6 weeks after AngII infusion was attenuated in C1qa-deficient mice compared with wild-type mice (Fig. 8g; Supplementary Fig. 6e,f), suggesting that C1-induced activation of β-catenin signalling, but not C1-induced activation of the classical complement cascade, mediates VSMC proliferation and hypertensive arterial remodelling.

## Discussion

**Figure 9:**
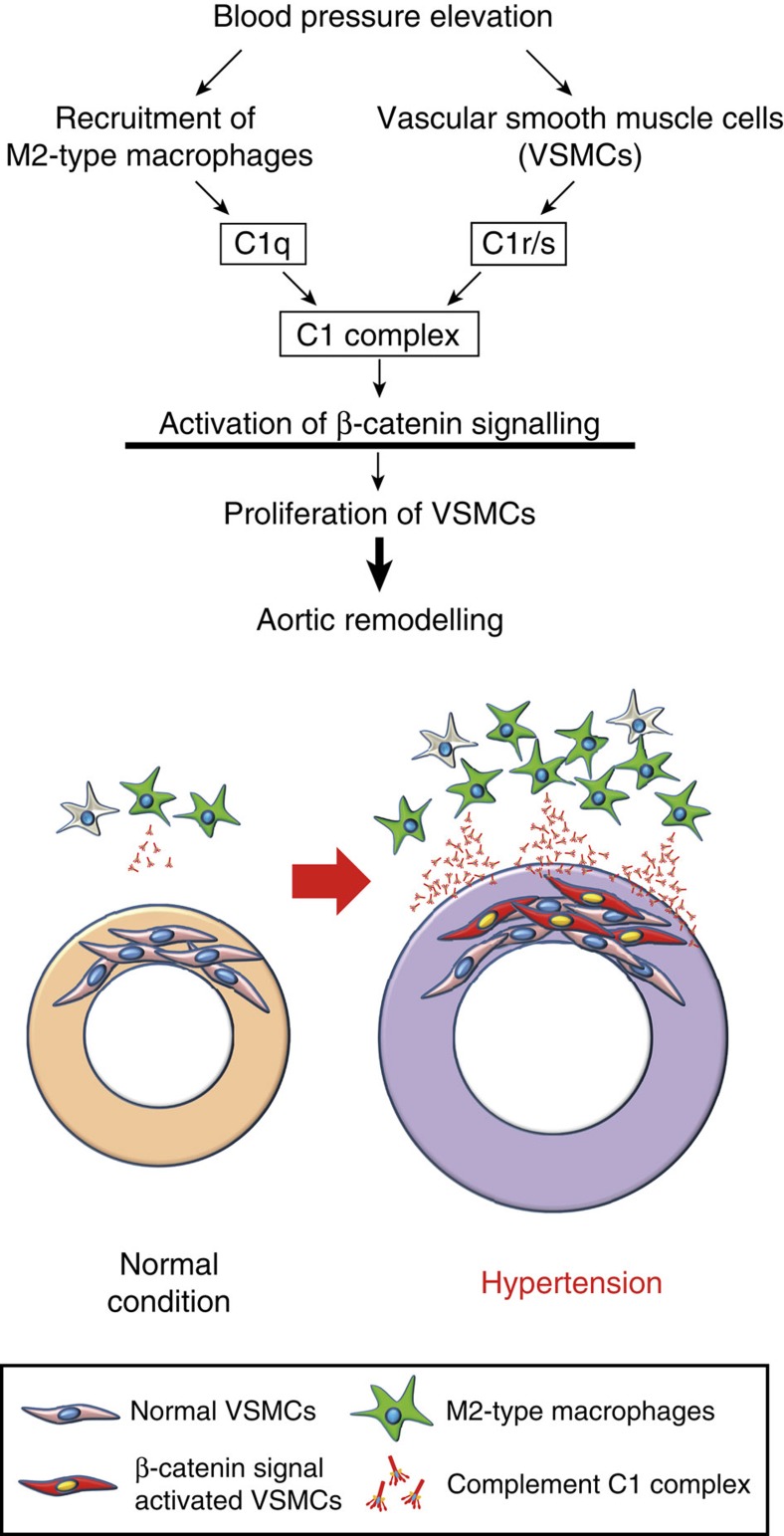
Mechanisms of hypertensive arterial remodelling. M2-type Mφs are recruited to the aortic adventitia after blood-pressure elevation and secrete C1q. Mφ-derived C1q and VSMC-derived C1r/s might compose the C1 complex, which plays a pivotal role in initiating hypertensive arterial remodelling through activating β-catenin signalling in VSMCs and inducing proliferation of VSMCs.

In this study, we have elucidated the novel molecular and cellular interplay that initiates hypertensive arterial remodelling. Mφs were recruited into the aortic adventitia soon after blood-pressure elevation and secreted complement C1q, which activated β-catenin signalling with C1r and C1s and induced proliferation of VSMCs, resulting in progression of hypertension-induced pathological arterial remodelling (Fig. 9).

It is well known that VSMC proliferation plays a key role in the progression of arterial remodelling in hypertension^31^ and in atherosclerosis^32^. Cell culture studies using hypertensive rats revealed the presence of cell autonomous and non-cell autonomous factors to explain the mitotic nature of VSMCs during hypertension^7^,^33^. Various growth factors and G-protein-coupled receptor agonists have been shown to induce proliferation of VSMCs in a non-cell autonomous manner. Most of these mitogenic stimuli activate mitogen-activated protein kinases, especially ERKs^6^,^20^. In the present study, AngII-induced activation of ERKs was not sufficient to promote VSMC proliferation *in vitro*. We also found no activation of ERKs in the aortic tissue 1 week after AngII infusion when the VSMC proliferation was already observed, suggesting that ERKs are not involved in VSMC proliferation at the initial stage of hypertensive arterial remodelling (Supplementary Fig. 1a–c).

Compared with the critical roles of Wnt/β-catenin signalling during embryonic angiogenesis^34^, its role during the postnatal period is less investigated. Activation of β-catenin signalling in intimal thickening has been reported^14^,^15^; however, these results have been derived from a specific situation such as after the acute ligation of the arteries or the direct mechanical injury to the arterial lumen. In the present study, we showed the activation of β-catenin signalling in a mice model of AngII-induced blood-pressure elevation, which is more physiological than previous reports. Inhibition of β-catenin signalling ameliorated the effect of high blood pressure on VSMC proliferation, suggesting the critical role of β-catenin signalling as a regulator of arterial remodelling during the postnatal period.

Vascular inflammation is a well-known pathogenic feature in arterial remodelling during hypertension and atherosclerosis^4^,^5^,^6^. A massive recruitment of Mφs to the adventitia of the aortic wall after AngII infusion was consistent with previous reports^35^,^36^. It remains unclear which type of Mφs is involved in hypertensive arterial remodelling. We elucidated that the anti-inflammatory (M2) phenotype but not pro-inflammatory (M1) phenotype was the prevailing characteristic of aortic Mφs that infiltrates into aortic adventitia soon after blood-pressure elevation (Fig. 6a,b). M2-polarized Mφs secreted a factor that induces proliferation of VSMCs by activating β-catenin signalling, and one of the potent candidates was C1q. We have reported that complement C1q activates β-catenin signalling through C1s-dependent enzymatic cleavage of LRP6 (ref. 16). Here, we revealed that M2-type Mφ-derived C1q and VSMC-derived C1r/s might compose the C1 complex and activate β-catenin signalling in VSMCs (Fig. 9).

In the previous report, we showed that C1q is the molecule that is increased in the blood by aging and activates β-catenin signalling. Here we first demonstrated that the C1 complex activates β-catenin signalling and induces proliferation of VSMCs during the early stage of hypertension, which leads to arterial remodelling at the later stage.

It was previously reported that C1-INH treatment blocked neointima formation following arterial injury in atherosclerotic mice^37^, indicating that C1q progresses injury-induced arterial remodelling. Conversely, Mφ infiltration into plaque and progression of atherosclerotic plaque formation in low-density lipoprotein receptor-deficient mice were exaggerated by additional *C1qa* gene disruption^38^. This evidence suggests that C1q might also mediate anti-inflammatory and anti-atherosclerotic effects in arterial plaque progression. Together with our findings, the role of C1 or C1q in arterial remodelling may differ depending on its aetiology. In addition, the genetic or pharmacologic loss-of-function experiments did not completely inhibit proliferation of VSMCs after AngII infusion, suggesting that pathways other than the C1-β-catenin pathway are also involved in this phenomenon.

Our findings provide a novel mechanistic link between humoral innate immunity and arterial remodelling, and suggest that blocking C1-induced activation of β-catenin signalling becomes a novel therapeutic strategy to prevent arteriosclerosis associated with hypertension.

## Methods

### Reagents

BrdU, tamoxifen and clodronate disodium were purchased from Sigma. Human complement C1q and C1 complexes were from Calbiochem. AngII and hydralazine hydrochloride were from Wako. C1-INH (Berinert) was from CSL Behring. PKF115-584 (ref. 22) was from Novartis. Human recombinant Wnt3A was from R&D. Mouse recombinant IL-4 was from PeproTech. Mouse monoclonal antibody (14/Beta-Catenin) against β-catenin was from BD Transduction Laboratories (immunofluorescence (IF) dilution 1:200). Rat monoclonal antibody (clone CI:A3-1) against mouse F4/80 (IF dilution 1:50), rabbit monoclonal antibody (clone E247) against β-catenin (WB dilution 1:2,000), rabbit polyclonal antibody against axin2 (WB dilution 1:2,000, IF dilution 1:100) and rat monoclonal antibody against BrdU (clone BU1 75(ICR1)) (IF dilution 1:200) were from Abcam. Mouse monoclonal antibody (clone 8E7) against ABC was from Millipore (WB dilution 1:1,000). Rabbit polyclonal antibody against actin and alpha-smooth muscle actin (αSMA) were from Sigma (IF dilution 1:200). TACS 2TdT Fluorescein Kit was from Trevigen. Mouse monoclonal antibodies against C1r (WB dilution 1:250) and C1s (WB dilution 1:250) were from R&D. Secondary antibodies conjugated to Alexa Fluor 488 and Alexa Fluor 546 were from Molecular Probes (IF dilution 1:200).

### Animals

Male mice 8–10 weeks of age were used for all experiments. C57BL/6 mice were purchased from CLEA Japan. Axin2^LacZ^ mice^11^ were from the Jackson laboratory. C1qa knockout mice^39^, C3 knockout mice^40^ and SMMHC-CreER^T2^ mice^23^ were previously described, and mice backcrossed into C57BL/6 background were used for experiments. Conditional β-catenin knockout mice (*Ctnnb1*^flox/flox^ mice) were established as previously described^41^. For AngII infusion, an osmotic minipump (Alzet) containing either saline or AngII (1.8 μg kg^−1^ min^−1^) was implanted subcutaneously. Blood pressure was measured in conscious mice by the tail-cuff system using BP98A (Softron) according to the manufacturer’s protocol. To induce Cre/loxP-mediated gene disruption in SMMHC-CreER^T2^ mice, tamoxifen dissolved in corn oil was injected intraperitoneally for 5 consecutive days (1 mg per day). Hydralazine was administered in drinking water (250 mg l^−1^) 1 week before the implantation of an osmotic minipump, and the solution was replaced every single day. Control mice received drinking water alone. Clodronate disodium was encapsulated into liposomes by Katayama Chemical Industries Co., Ltd (Osaka, Japan). Clodronate liposomes were prepared by freeze–thawing and filter extrusion. Dipalmitoylphosphatidylcholine, cholesterol and dipalmitoylphosphatidylserine were mixed at the molar ratio 50:40:10. The dry lipid mixture was solubilized in PBS or clodronate. The resulting vesicles were freeze–thawed in liquid nitrogen and water at 40°C, followed by filter extrusion through 400-nm membranes (Nuclepore, Sterico, Dietikon, Switzerland) using the Lipex extruder (Lipex Biomembranes Inc., Vancouver, Canada). The suspension was ultrafiltrated using PBS through an Amicon XM300 membrane to remove free clodronate. The size of liposome was measured by dynamic light-scattering spectrophotometry (Zetasizer Nano-ZS, Malvern, Worcestershire, UK) at 25°C. Clodronate liposomes contain ~10 mg clodronate per ml and have a mean diameter of 250±50 nm. PBS liposomes or clodronate liposomes (200 μl) were administered intravenously 3 days before the implantation of an osmotic minipump and every 3 days thereafter. Dimethylsulphoxide (DMSO) or PKF115-584 (0.16 mg kg^−1^) was administered every other day via intraperitoneal injections from 5 days before the implantation of an osmotic minipump. PBS or C1-INH (15 U per day) was administered intravenously 5 days before the implantation of an osmotic minipump and every other day thereafter. All experiments were approved by the University of Tokyo Ethics Committee for Animal Experiments and strictly adhered to the guidelines for animal experiments of the University of Tokyo.

### Cell culture

HASMCs were cultured in smooth muscle growth medium-2 (Lonza), which contains fetal bovine serum (FBS) and various growth factors, and starved for 24 h before stimulation in smooth muscle basal medium-2 (Lonza) devoid of serum or growth factors. Raw264.7 cells were purchased from American Type Culture Collection and cultured in Dulbecco’s modified Eagle’s medium supplemented with 10% FBS. BMDMs were isolated from femurs of wild-type mice and cultured in Mφ medium (RPMI 1640 medium supplemented with 10% FBS, 40 ng ml^−1^ murine Macrophage Colony-Stimulating Factor (M-CSF), 2 mM L-glutamine, 50 U ml^−1^ penicillin and 100 μg ml^−1^ streptomycin). For M1 or M2 polarization, Raw264.7 cells or BMDMs were treated with LPS (50 ng ml^−1^) or IL-4 (20 ng ml^−1^) for 24 h, respectively. CM of Raw264.7 cells or BMDMs were collected after another 24 h.

### RNA analysis

Total RNA was extracted using TRIzol reagent (Invitrogen) according to the manufacturer’s instructions. RNA was treated with DNase and reverse transcribed using the QuantiTect Reverse Transcription Kit (Qiagen). Real-time quantitative PCR was performed using the Universal Probe Library (UPL) (Roche) and Light Cycler TaqMan Master kit (Roche). Relative levels of gene expression were normalized to the *Gapdh* gene expression using the comparative Ct method. Primer sequences and the corresponding UPL numbers were designed using an online program provided by Roche. Primer sequences are provided in Supplementary Table 1.

### Protein analysis

Abdominal aorta (between the diaphragm and the left renal artery) was minced and lysed in buffer containing 20 mM HEPES (pH: 7.9), 150 mM NaCl, 5 mM EDTA, 15% glycerol, 1% Triton X-100, a protease inhibitor cocktail and phosphatase inhibitor cocktail. Total cell lysate of the cultured cells was lysed in the same buffer. Cytosolic fraction of the cultured cells was obtained using ultracentrifuge. Culture media was concentrated using Amicon Ultra 30 K (Millipore). The proteins were fractioned using 8–10% SDS–polyacrylamide gel electrophoresis and analysed using immunoblotting. Densitometry analysis on the bands was calculated using ImageJ. The non-cropped blots for the representative images are displayed in Supplementary Fig. 7.

### Cell proliferation

In cell culture experiments, cells were labelled by adding BrdU (10 μM) to the culture media for 12 h. Cells were then immunostained with anti-BrdU antibody. The percentage of BrdU-positive cells in nine randomly chosen low power fields were calculated for each sample. In mice experiments, BrdU (100 mg kg^−1^) was injected intraperitoneally 24 h before euthanization. Dissected aortic tissues were embedded in Tissue-Tek® O.C.T.™ (Optimal Cutting Temperature) Compound (SAKURA) and sectioned at 5 μm thickness. After immunostaining with anti-BrdU (1:200) and anti-αSMA (1:200) antibodies, the number of double-positive (BrdU(+)/αSMA(+)) cells per each section was counted. Six sections were examined for each animal and the mean number was shown.

### Histological analysis

For morphological analysis, aortic tissues were fixed with formaldehyde and embedded in paraffin. For fluorescent immunostaining, 5-μm-thick fresh-frozen sections were stained and the nuclei were counterstained with 4′,6-diamidino-2-phenylindole. Images were acquired using the LSM510 or LSM700 confocal microscope (Zeiss) or FSX100 (Olympus) and analysed using ImageJ.

### Flow cytometric analysis of the aortic tissue

Aortic tissues were minced and digested in digestion solution containing Elastase (Worthington) (0.25 mg ml^−1^) and LiberaseTH (Roche) (0.025 mg ml^−1^). Digested tissues were further dissociated with a 21-G needle. Remaining deposited debris was removed and the supernatant was collected after filtering through a 40-μm cell strainer. Cells were suspended in PBS containing 3% FBS, and nonspecific binding of the antibodies to Fc receptors was blocked using an Fc receptor-blocking agent (1:50) (BioLegend). Cells were stained with APC-anti-mouse CD11b (1:150), PE anti-mouse F4/80 (1:20), APC-Cy7 anti-mouse Ly6c (1:300) and Alexa488 anti-mouse CD206 (1:50) (BioLegend). The LIVE/DEAD Fixable Aqua Dead Cell Stain Kit (Invitrogen) was used to label dead cells. After washing, cells were analysed using BD FACSVerse. Cell sorting was performed by BD FACSAria II. The data were analysed by Flo Jo software (Tree Star).

### Statistical analysis

All values are reported as mean±s.d. Statistical calculations were performed using GraphPad Prism 6 (GraphPad software Inc.). We analysed the data using the unpaired two-tailed Student’s *t*-test (parametric) or the unpaired two-tailed Mann–Whitney *U*-test (non-parametric) in case of analysing two groups. The one-way analysis of variance with Turkey’s *post hoc* test (parametric) or the Kruskal–Wallis test with Dunn’s correction for multiple comparisons (non-parametric) was used in case of analysing multiple groups. The two-way analysis of variance followed by Sidak’s multiple comparisons test was used to compare the effect of multiple levels of two factors. The *F*-test, Browne–Forsythe test or the Bartlett’s test was used to determine the distributional assumption (normality and homogeneity of variance) of the data. When the data do not fit a normal distribution, non-parametric tests are used. Significant differences were defined as *P*<0.05.

## Author contributions

I.K. planned and supervised the project. T.Su., A.T.N., I.S. and I.K. designed the experiments. T.Su., S.N., T.H., A.N., M.I. and T.Y. performed the experiments. K.O., T.Sa., A.H., M.I. and T.Y. analysed data. S.O., T.No. and M.B. contributed new reagents/analytical tools. H.A., T.O., J.-K.L., T.M., Y.K., H.M., I.M. and T.Na. advised on the experiments. T.Su., A.T.N., I.S. and I.K. wrote the manuscript.

## Additional information

**How to cite this article:** Sumida, T. *et al*. Complement C1q-induced activation of β-catenin signalling causes hypertensive arterial remodelling. *Nat. Commun.* 6:6241 doi: 10.1038/ncomms7241 (2015).

## Supplementary Material

Supplementary Information Supplementary Figures 1-7, Supplementary Table 1.
