# Supplementary material for: Complement C1q-induced activation of β-catenin signalling causes hypertensive arterial remodelling
Source: Nat Commun. 2015 Feb 26;6:6241. doi: 10.1038/ncomms7241 (PMC4351572; doi:10.1038/ncomms7241)
Supplement: Supplementary Information — Supplementary Figures 1-7, Supplementary Table 1. [file ncomms7241-s1.pdf]

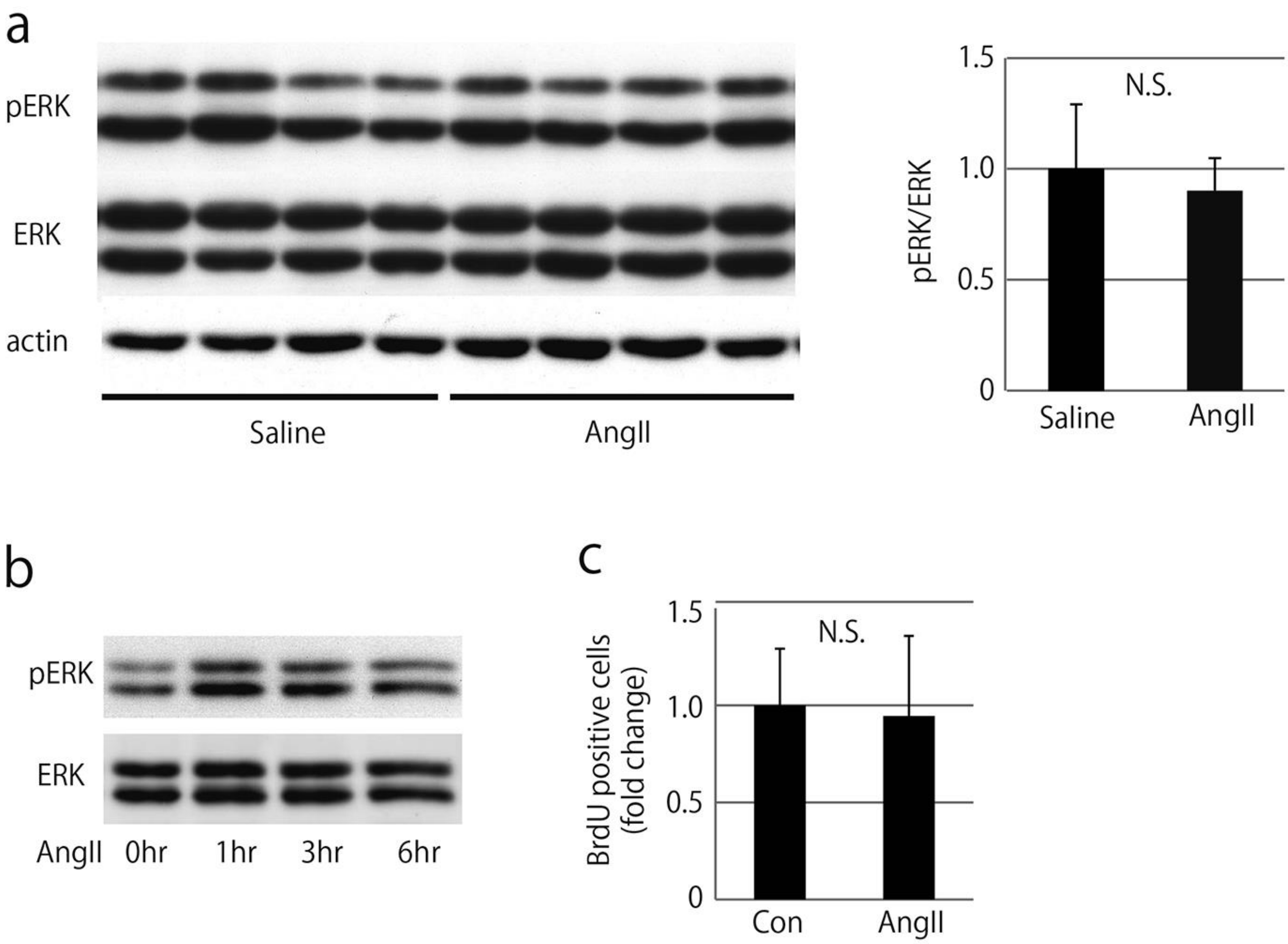

Supplementary Figure 1. ERK signaling is not activated at early hypertension.

a, Western blot analysis for the level of phospho-ERK (pERK) and total ERK in the aortic tissue from the saline- or AngII-infused mice. The ratio of pERK and total-ERK is shown (n=7). N.S.; not significant. **b,** Western blot analysis for pERK and total ERK in HASMCs treated with AngII (2 μM). **c,** The number of BrdU positive HASMCs after AngII (2 μM) treatment. (n=4). Statistical significance was determined using the unpaired two-tailed Student's t-test for **a, c.** Results are represented as mean ± SD.

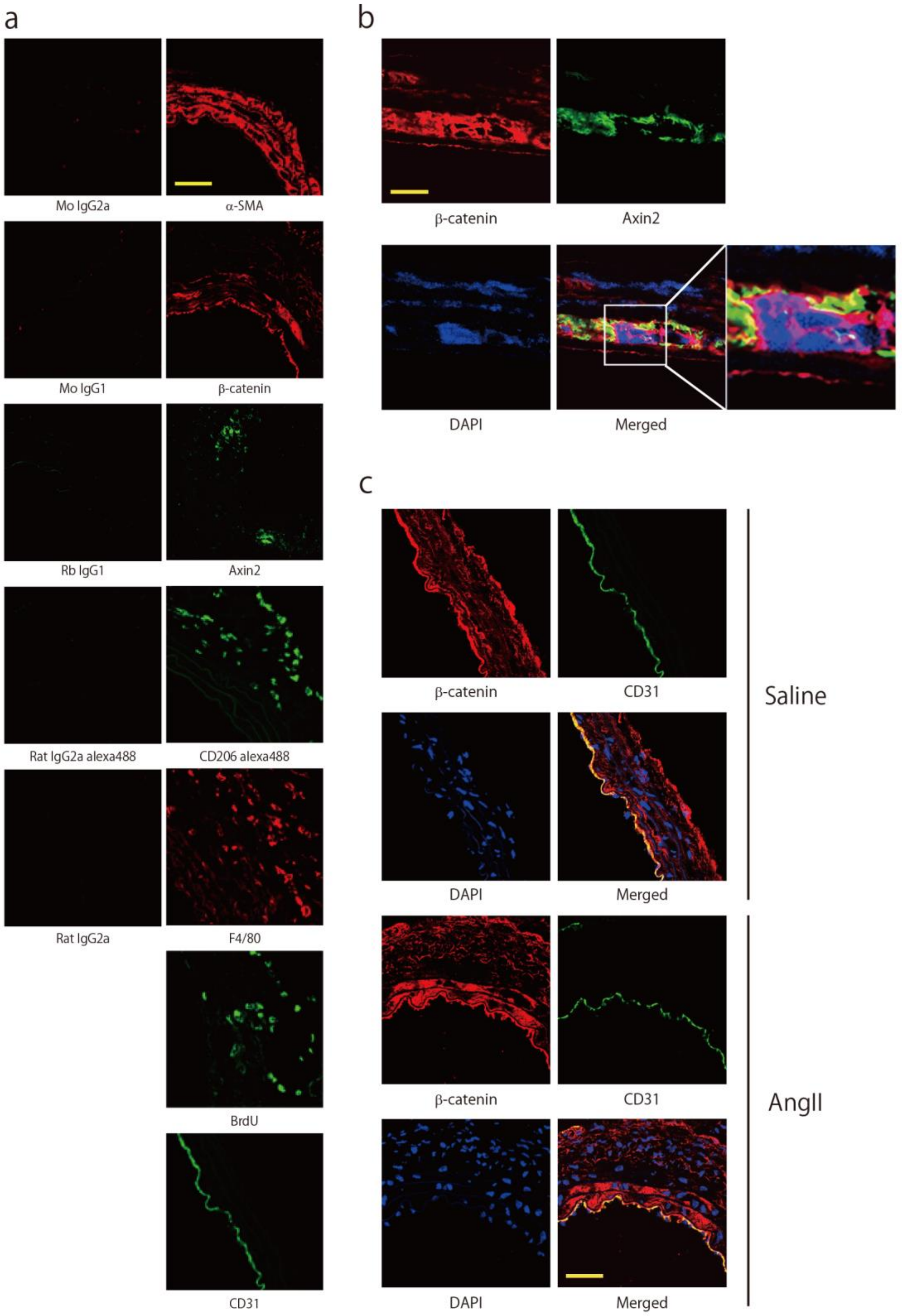

Supplementary Figure 2. β-catenin signaling is activated in aortic media after AngII-infusion.

a, Left, immunofluorescence staining of aortic tissues from 1 week AngII-infused mice using isotype control antibody. Right, immunofluorescence staining of aortic tissues from AngII-infused mice using antigen specific antibodies: αSMA, β-catenin, Axin2, CD206, F4/80, BrdU, and CD31. The images immunostained with each corresponding isotype control are shown on the left side. The IgG isotype of anti-F4/80 antibody, anti-BrdU antibody, and anti-CD31 antibody were the same. Scale bar, 50μm. **b,**

Immunostaining of aortic tissue from 1 week AngII-infused mice for β-catenin (red), Axin2 (green), and DAPI (blue). The magnified view shows the co-localization of β-catenin and DAPI in aortic media indicating the nuclear translocation of β-catenin. Scale bar, 20μm. **c,** Aortic tissues from 1 week saline- or AngII-infused mice were immunostained for CD31 (green) and β-catenin (red). Scale bar, 50 μm.

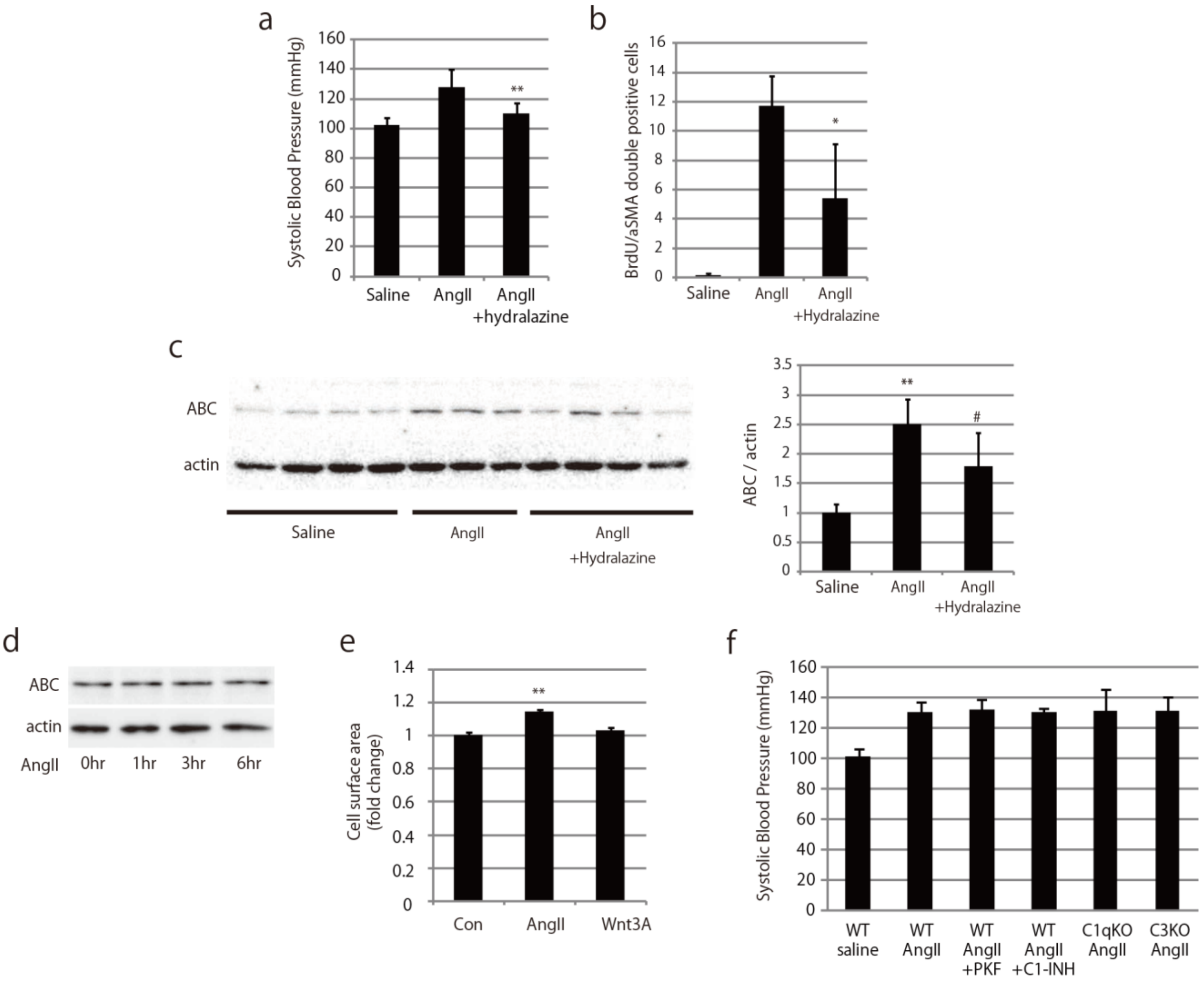

Supplementary Figure 3. High blood pressure partly contributes to activation of β-catenin signaling.

a, Increased systolic blood pressure after 1week AngII-infusion was lowered by hydralazine treatment. **p<0.01 versus AngII-infused mice treated with PBS (AngII). **b,** The number of double positive (BrdU(+)/αSMA(+)) cells per section from 1 week saline-infused mice (Saline), 1 week AngII-infused mice treated with PBS (AngII), or with hydralazine (AngII+hydralazine). *p<0.05 versus 1 week AngII-infused mice treated with PBS (n=6-7). **c,** Western blot analysis for non-phosphorylated active β-catenin (ABC) in the aortic tissues from 1 week saline-infused mice (Saline), 1 week AngII-infused mice treated with PBS (AngII), or 1 week AngII-infused mice treated with hydralazine (AngII+hydralazine). Densitometric analysis for the amount of ABC in the aortic tissue from each group is shown. Activation of β-catenin signaling was quantified by measuring the relative level of ABC over actin. The values are shown as fold induction over saline-infused mice. **p<0.01 versus saline-infused mice, #p<0.05 versus AngII-infused mice (n=7). **d,** Western blot analysis. HASMCs were treated with AngII (2 μM) and the amount of ABC was analyzed. **e,** Cell surface area of HASMCs was measured using ImageJ after AngII (2 μM) or Wnt3A (80 ng/ml) treatment. The values are shown as fold induction over non-treated HASMCs (Con). **p <0.01 versus non-treated HASMCs (n=3). **f,** Systolic blood pressure after 1week AngII-infusion. PKF or C1-INH treatment did not affect

blood pressure. C1qKO or C3KO mice showed blood pressure levels similar to wild type mice. Statistical significance was determined using the one-way ANOVA with Turkey's post hoc test for **a-c, e, f**. Results are represented as mean ± SD.

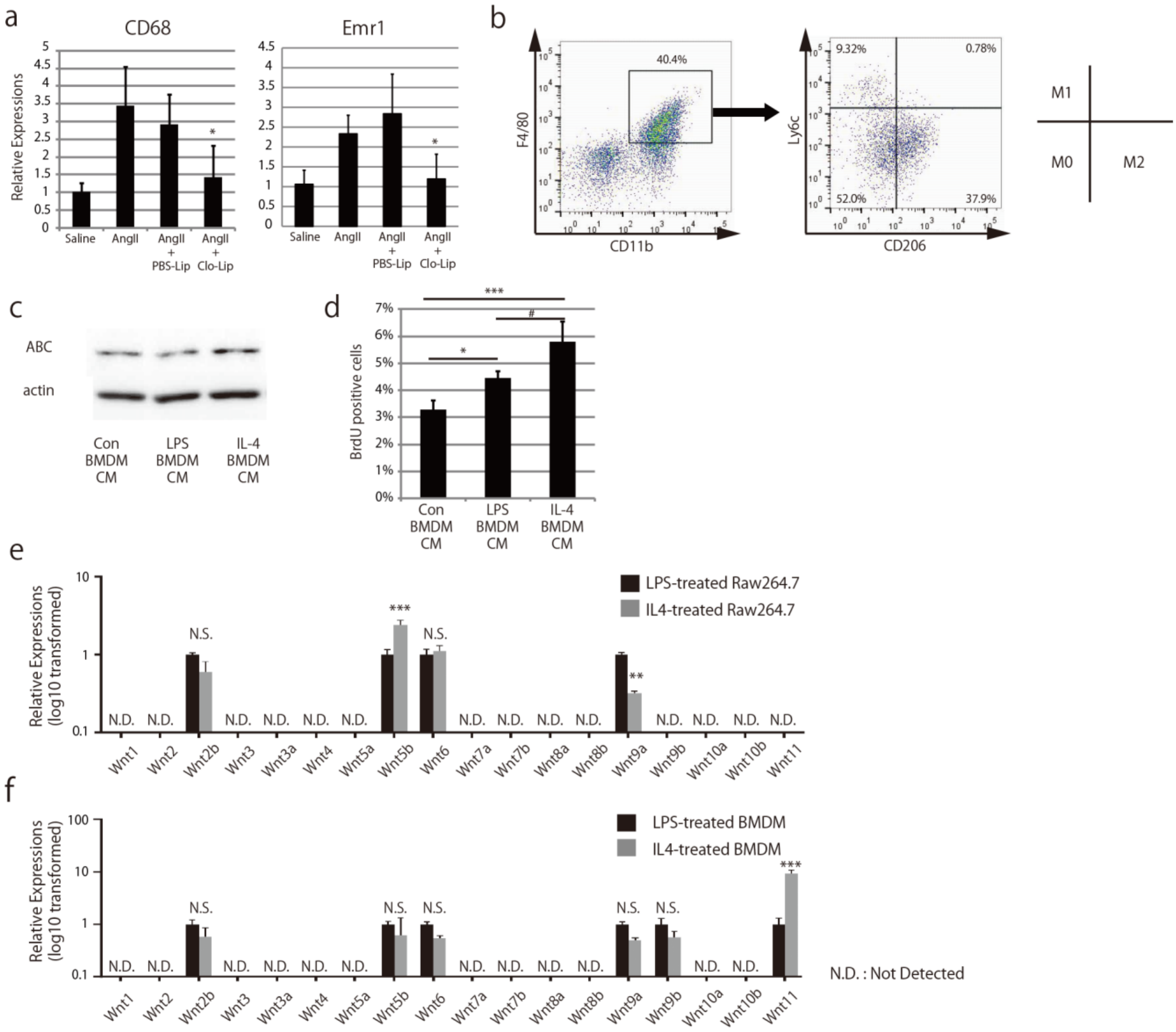

Supplementary Figure 4. Aortic M2-type Mϕ activates β-catenin signaling.

a, Expression levels of Mϕ markers (*CD68* and *Emr1*) in the aortic tissues from saline- or AngII-infused mice were analyzed by real-time PCR. The values are shown as fold-induction over saline-infused mice. *$p<0.05$ versus PBS-Lip treated AngII-infused mice (n=3; saline group, n=6; other groups). **b,** Representative density plots of aortic Mϕs isolated from AngII-infused mice. CD11b+F4/80+ Mϕs were divided into M0-type (CD11b+F4/80+CD206-Ly6c-), M1-type (CD11b+F4/80+CD206-Ly6c+), and M2-type (CD11b+F4/80+CD206+Ly6c-) Mϕs. **c,** Representative western blot analysis. Conditioned media from BMDMs treated with PBS (Con BMDM CM), LPS (50 ng/mL) (LPS BMDM CM), or IL-4 (20 ng/mL) (IL-4 BMDM CM) were added to HASMCs. The amount of ABC in the total cell lysate of HASMCs was analyzed. **d,** HASMCs were treated as in **c,** and the percentage of BrdU positive cells was calculated. *$p<0.05$, ***$p<0.001$ versus Con BMDM CM, $^{\#}p<0.05$ versus LPS BMDM CM (n=4). **e, f,** Real-time PCR analyses for the expression of the all Wnt ligands in Mϕs (Raw 264.7 cells or BMDMs). RNA was extracted from Raw264.7 cells or BMDMs treated with LPS (50 ng/ml) or IL-4 (20 ng/mL) for 24 hours. The values are shown as fold induction over LPS-treated Mϕs. **$p<0.01$, ***$p<0.001$ versus LPS-treated Raw 264.7 cells or

BMDMs (n=3). N.S.; not significant. N.D., Not detected. Statistical significance was determined using the one-way ANOVA with Turkey's post hoc test for **a, d,** and the two way ANOVA followed by Sidak's multiple comparisons test for **e, f.** Results are represented as mean ± SD.

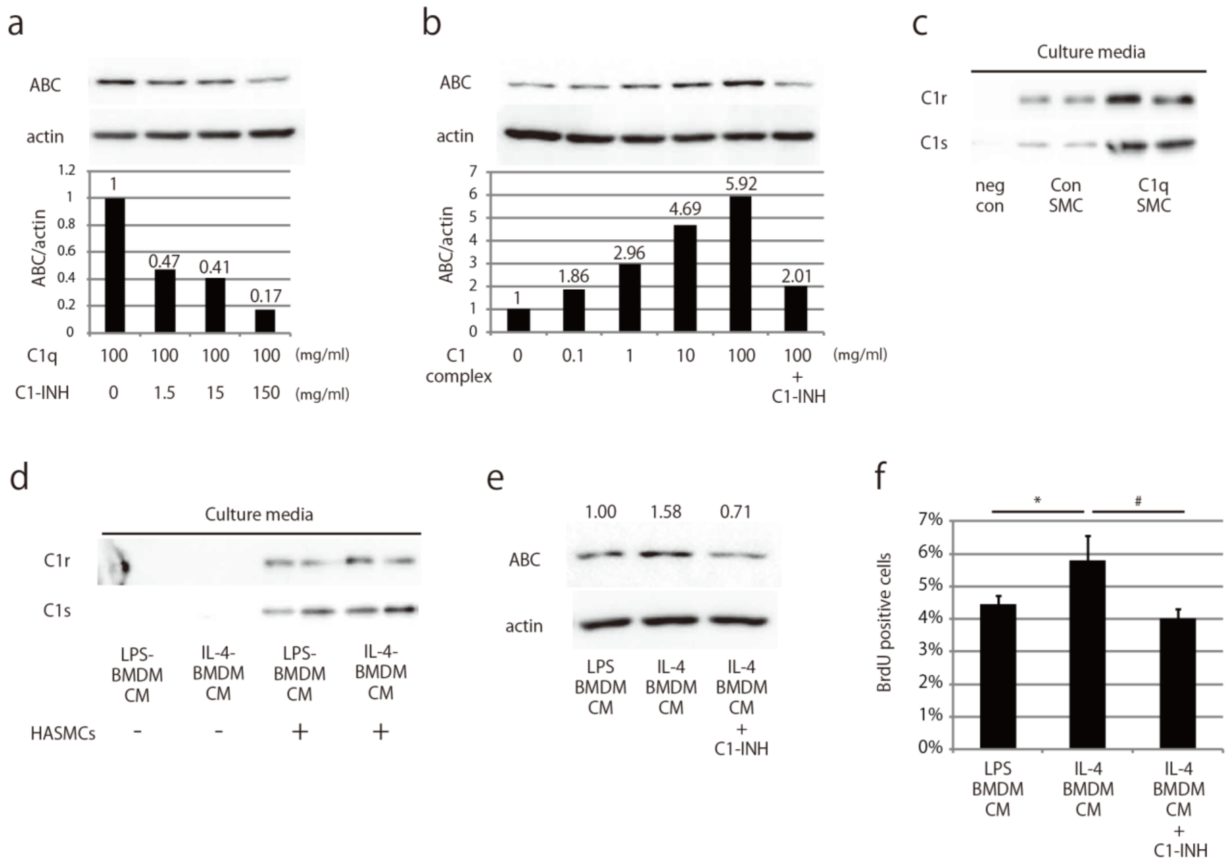

Supplementary Figure 5. C1 complex activates β-catenin signaling in VSMCs.

a, **b,** Representative western blot analysis. **a:** HASMCs were treated with C1q (100 μg/ml) and C1-INH (0, 1.5, 15, 150μg/ml), **b:** HASMCs were treated with C1 complex (0, 0.1, 1, 10, 100 μg/ml) and C1INH (150 μg/ml). The protein amount of ABC was analyzed by western blotting (upper panel) and the relative band intensity of ABC is shown in the bar graph after normalization for the level of actin (lower panel). **c,** HASMCs were cultured and stimulated with or without C1q (100 μg/ml) in a serum-free condition for 24h. Culture media were concentrated and analyzed by western blotting. Culture media incubated without HASMCs were shown as negative control (negcon). **d,** Serum-free conditioned media from BMDMs treated with LPS (50 ng/ml) (LPS BMDM CM) or IL-4 (20 ng/mL) (IL-4 BMDM CM) were added into the culture dish with or without HASMCs for 24h. Culture media were concentrated and analyzed by western blotting. **e,** Representative western blot analysis. LPS BMDM CM or IL-4 BMDM CM were added to HASMCs with or without C1-INH (150 μg/ml). The protein amount of ABC in the total cell lysate of HASMCs was analyzed and the relative intensity of each band is shown over each immunoblot after normalization for the level of actin. **f,** HASMCs were treated as in **e,** and the number of BrdU positive cells was counted. *$p<0.05$ versus LPS BMDM CM, $^{\#}p<0.05$ versus IL-4 BMDM CM (n=4). Statistical significance was determined using the one-way ANOVA with Turkey's post hoc test for **f**. Results are represented as mean ± SD.

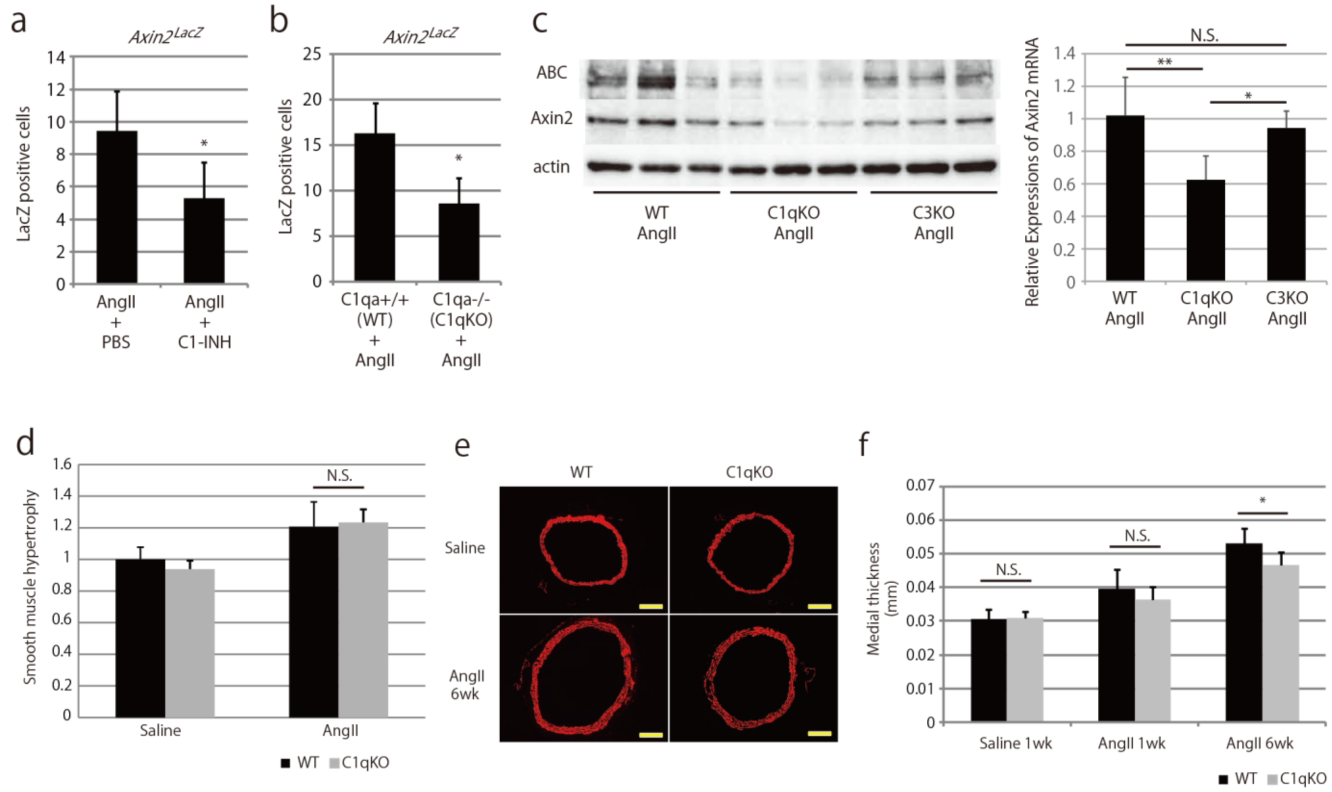

Supplementary Figure 6. C1qa deletion attenuates AngII-induced arterial remodeling.

a, The number of LacZ positive cells in the media of aortic tissues from 1 week AngII-infused Axin2^{LacZ} mice treated with PBS or with C1-INH is shown. *p<0.05 versus AngII-infused Axin2^{LacZ} mice treated with PBS (n=5). **b,** The number of LacZ positive cells in the media of aortic tissues from 1 week AngII-infused *Axin2*^{LacZ}*/C1qa*$^{+/+}$ (Axin2^{LacZ}/WT) mice or *Axin2*^{LacZ}*/C1qa*$^{-/-}$ (Axin2^{LacZ}/C1qKO) mice is shown. *p<0.05 versus AngII-infused Axin2^{LacZ}/WT mice (n=4). **c,** Representative western blot analysis for ABC and Axin2 (left panel) and real-time PCR for the expression level of *Axin2* (right panel) in the aortic tissues from 1 week AngII-infused wild type mice (WT AngII), C1qa-deficient mice (C1qKO AngII), and C3-deficient (C3KO AngII) mice. **p<0.01 versus WT AngII mice, *p<0.05 versus C1qKO AngII mice, N.S.; not significant. (n=4-6). **d,** Morphometric analysis of smooth muscle hypertrophy. Aortic tissues were stained for αSMA and DAPI. Hypertrophic change of the smooth muscle cells was calculated by dividing αSMA-positive area with the number of nuclei. The values are shown as fold induction over saline-infused WT mice. (n=6-7). **e,** Immunostaining for αSMA of the abdominal aorta from 6 weeks saline-infused mice (Saline 6wk) and 6 weeks AngII-infused mice (AngII 6wk). Scale bar, 200 μm. **f,** Morphometric analysis. Media thickness was calculated using ImageJ. *p<0.05 versus 6 week AngII-infused WT mice (n=5-7). Statistical significance was determined using the unpaired two-tailed Student’s t-test for **a, b,** the one-way ANOVA with Turkey’s post hoc test for **f**, and the two way ANOVA followed by Sidak’s multiple comparisons test for **d, f.** Results are represented as mean ± SD.

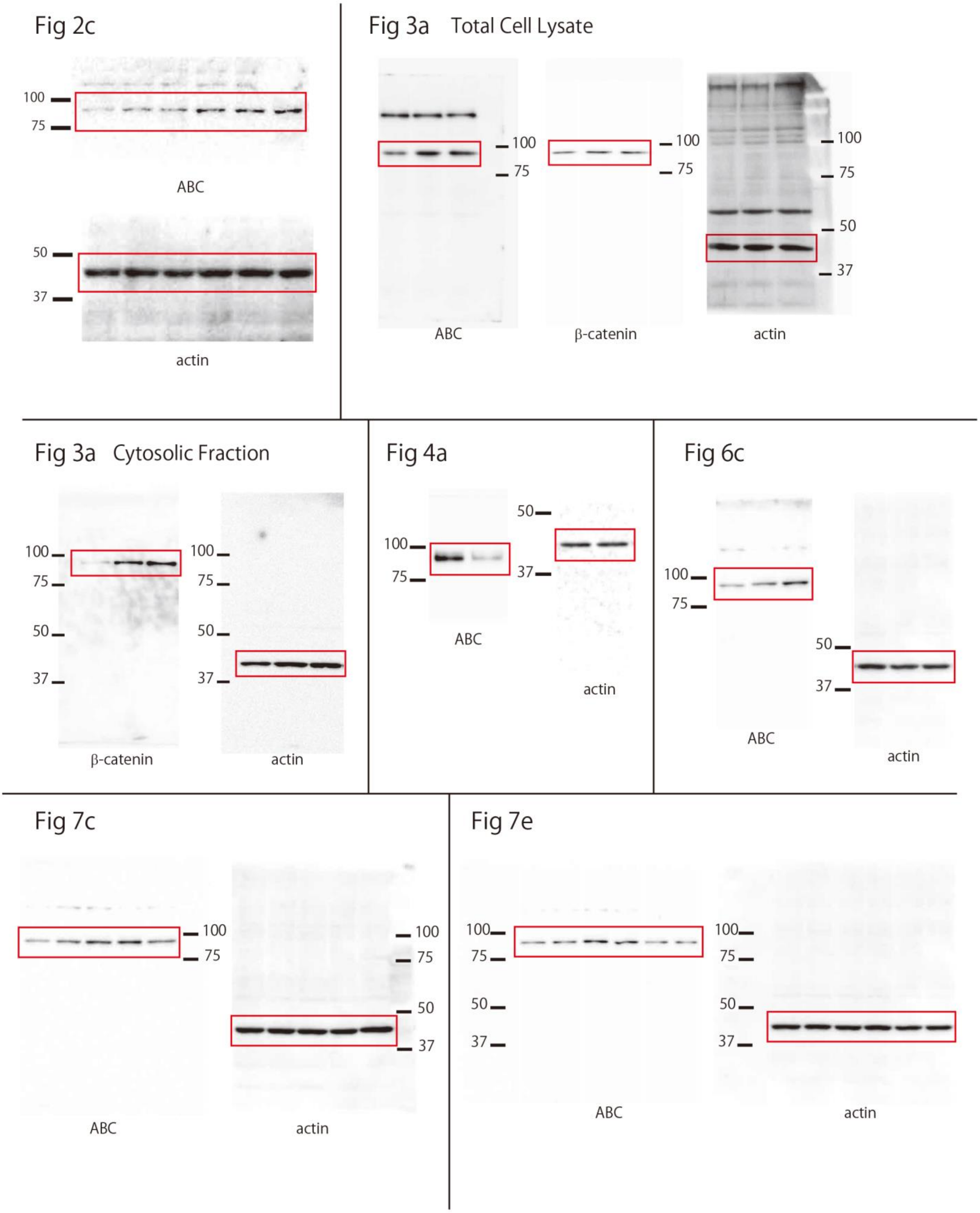

Supplementary Figure 7. Full images of western blots.

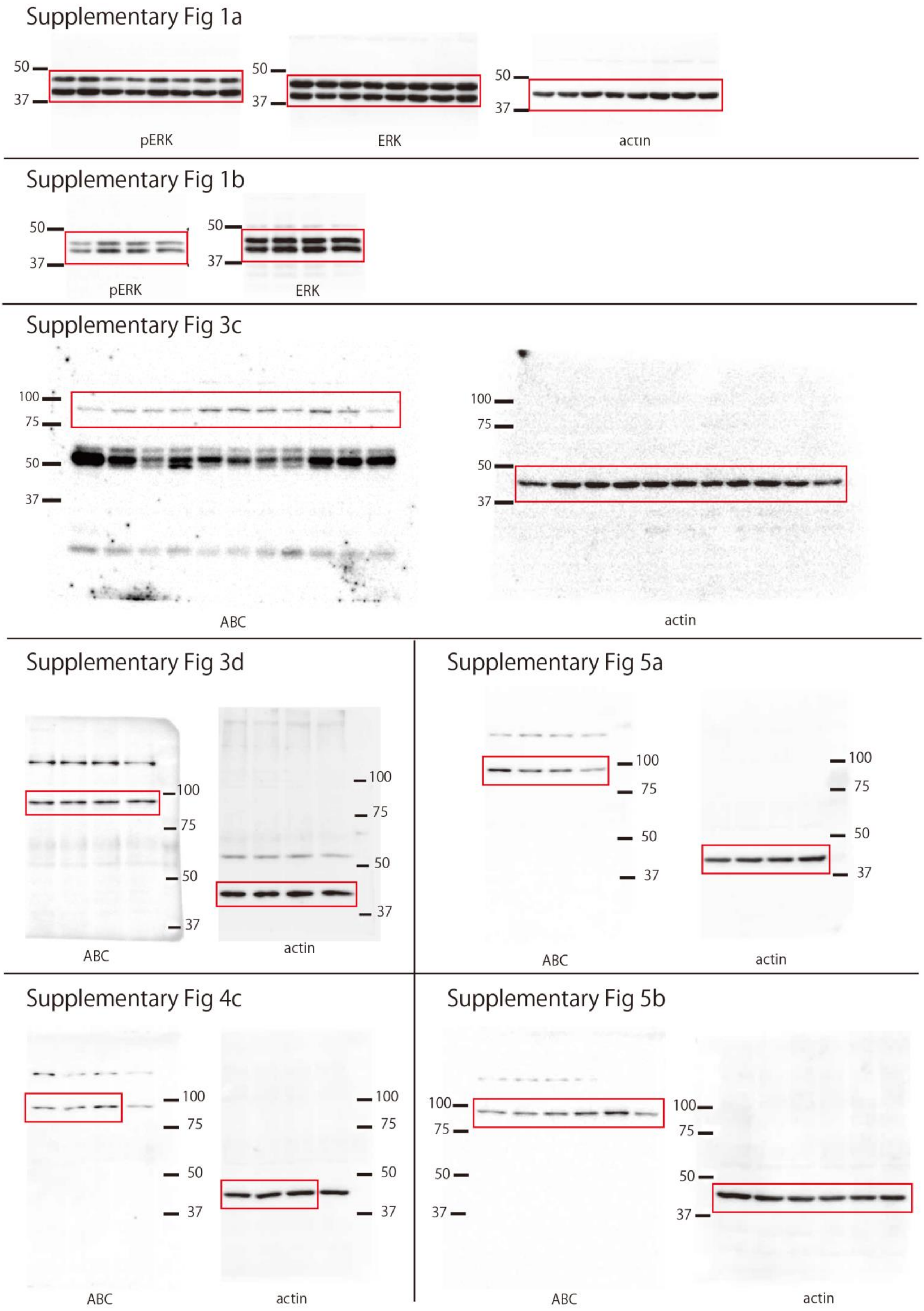

Supplementary Figure 7 (Continued). Full images of western blots.

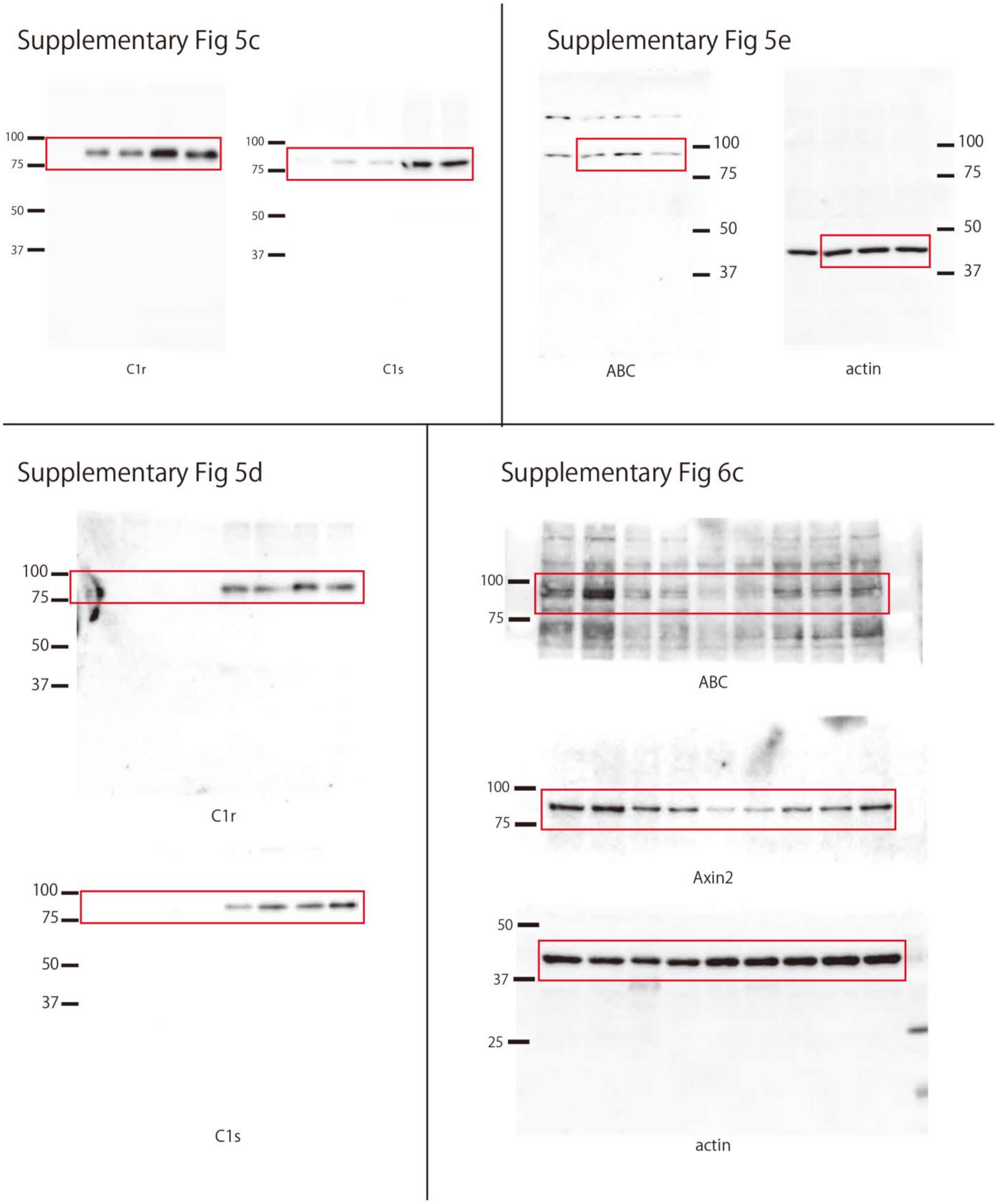

Supplementary Figure 7 (Continued). Full images of western blots.
Blotting membrane for Supplementary Fig 4c and Supplementary Fig 5e is the same.

Supplementary Table1. Primer sequences used for qRTPCR.

GAPDH	Fwd	tgtccgtcgtggatctgac
	Rev	cctgcttcaccaccttcttg
C1qa	Fwd	cgggtctcaaaggagagaga
	Rev	ttaaaacctcggataccagtcc
C1ra	Fwd	aaccatattacaagatgctgacca
	Rev	ccttgggctgtgcaggta
C1s	Fwd	ggtggatacttctgctcctgtc
	Rev	agggcagtgaacacatctcc
Wnt1	Fwd	tactggcactgaccgctct
	Rev	cttggaatccgtcaacaggt
Wnt2	Fwd	cctgatgaaccttcacaacaac
	Rev	tcttgtttcaagaagcgctttac
Wnt2b	Fwd	ccgggaccacactgtcttt
	Rev	gctgacgagatagcatagacga
Wnt3	Fwd	ctcgctggctacccaattt
	Rev	gaggccagagatgtgtactgc
Wnt3a	Fwd	cttagtgctctgcagcctga
	Rev	gagtgctcagagaggagtactgg
Wnt4	Fwd	actggactccctccctgtct
	Rev	tgcccttgtcactgcaaa
Wnt5a	Fwd	acgcttcgcttgaattcct
	Rev	aatattccaatgggcttcttcat
Wnt5b	Fwd	agcaccgtggacaacacat
	Rev	aaggcagtctctcggctacc
Wnt6	Fwd	gtgcaactgcacaacaacg
	Rev	ggaacggaggcagcttct
Wnt7a	Fwd	cgctgggagagcgtactg
	Rev	cgataatcgcataggtgaagg
Wnt7b	Fwd	gcgtcctctacgtgaagctc
	Rev	tcttgttgcagatgatgttgg
Wnt8a	Fwd	tgacgagtcacaaaatggaaa
	Rev	cccgaactccacgttgtc
Wnt8b	Fwd	gcagcctcggagactttg
	Rev	ctccccagagccaacctt
Wnt9a	Fwd	cgagtggacttccacaacaa
	Rev	ggcatttgcaagtggtttc
Wnt9b	Fwd	ccaagagaggaagcaaggac
	Rev	tctcaggccgctcttcac

Wnt10a	Fwd	ggcgctcctgttcttccta
	Rev	gatgtcgttgggtgctgac
Wnt10b	Fwd	aatgcggatccacaacaac
	Rev	ctccaacaggtcttgaattgg
Wnt11	Fwd	ctgcatgaagaatgagaaggtg
	Rev	actgccgttggaagtcttgt
Axin2	Fwd	gagagtgagcggcagagc
	Rev	cggctgactcgttctcct
CD68	Fwd	gacctacatcagagcccgagt
	Rev	cgccatgaatgtccactg
Emr1	Fwd	cctggacgaatcctgtgaa
	Rev	tcccagagtgttgatgcaaa
CyD1	Fwd	agaaggagattgtgccatcc
	Rev	ctcttcgcacttctgctcct
β-TrCP	Fwd	gattatggacccggcagag
	Rev	agacctgggcatagagcaca
Wisp1	Fwd	tgttggacaccagagcctttt
	Rev	tgttggacaccagagcctttt
Wisp2	Fwd	cccactgatccatcttctgg
	Rev	tgtccaaggacaggcacag
